# The TissueTractor: A Device for Applying Large Strains to Tissues and Cells for Simultaneous High‐Resolution Live Cell Microscopy

**DOI:** 10.1002/smtd.202500136

**Published:** 2025-03-09

**Authors:** Jing Yang, Emily Hearty, Yingli Wang, Deepthi S. Vijayraghavan, Timothy Walter, Sommer Anjum, Carsten Stuckenholz, Ya‐Wen Cheng, Sahana Balasubramanian, Yicheng Dong, Adam V. Kwiatkowski, Lance A. Davidson

**Affiliations:** ^1^ Department of Bioengineering University of Pittsburgh Pittsburgh PA 15213 USA; ^2^ Department of Cell Biology University of Pittsburgh School of Medicine Pittsburgh PA 15213 USA; ^3^ Department of Computational and Systems Biology University Pittsburgh PA 15213 USA; ^4^ Department of Developmental Biology University of Pittsburgh Pittsburgh PA 15213 USA

**Keywords:** cytoskeletal remodeling, high‐resolution microscopy, live‐cell imaging, mechanosensors, mechanotransduction, tissue stretchers

## Abstract

Mechanical strain substantially influences tissue shape and function in various contexts from embryonic development to disease progression. Disruptions in these processes can result in congenital abnormalities and short‐circuit mechanotransduction pathways. Manipulating strain in live tissues is crucial for understanding its impact on cellular and subcellular activities, unraveling the interplay between mechanics and cells. Existing tools, such as optogenetic modulation of strain, are limited to small strains over limited distances and durations. Here, a high‐strain stretcher system, the TissueTractor, is introduced to enable simultaneous high‐resolution spatiotemporal imaging of live cells and tissues under strain applications varying from 0% to over 100%. We use the system with organotypic explants from *Xenopus laevis* embryos, where applied tension reveals cellular strain heterogeneity and remodeling of intracellular keratin filaments. To highlight the device's adaptability, the TissueTractor is also used to study two other mechanically sensitive cell types with distinct physiological roles: human umbilical vein endothelial cells and mouse neonatal cardiomyocytes, revealing cell morphological changes under significant strain. The results underscore the potential of the TissueTractor for investigating mechanical cues that regulate tissue dynamics and morphogenesis.

## Graphical abstract

Mechanical strain influences tissue development, homeostasis, and disease. The TissueTractor, a high‐strain stretcher system, enables high‐resolution imaging of live tissues and cells under strain, revealing cellular strain heterogeneity, cytoskeletal remodeling, and morphological adaptations in various model systems.

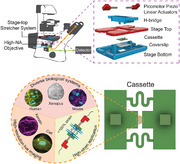

## 1 Introduction

Physical mechanics plays a critical role in orchestrating tissue shape and subsequent function across a range of contexts, including early embryonic development and later organogenesis, carcinogenesis, wound healing, and regeneration.^[^ ^1^, ^2^, ^3^, ^4^, ^5^, ^6^, ^7^, ^8^, ^9^, ^10^, ^11^, ^12^ ^]^ Mechanical strain, induced both by local microenvironmental and external sources, can guide biological processes such as cell migration, proliferation, cell fate change, etc.^[^ ^8^, ^9^, ^13^, ^14^, ^15^, ^16^, ^17^ ^]^ For instance, externally‐applied compressive forces can reduce cancer cell proliferation and induce apoptosis,^[^ ^11^, ^18^ ^]^ and in other cases, compressive forces can guide growth cone migration and drive collective neural crest cell migration.^[^ ^17^, ^19^ ^]^

Lesions to mechanical processes may lead directly to birth defects via inappropriate strain and tissue malformation or by altering normal mechanically‐triggered biological processes. These mechanical lesions can lead to structural birth defects, such as congenital heart defects, spina bifida, or ventral body wall closure.^[^ ^8^, ^17^, ^20^ ^]^ For instance, changes in embryo bulk mechanical properties can facilitate or delay the mesenchymal‐to‐epithelial transitions in *Xenopus* heart progenitor cells. These mechanical changes can cause cardiac defects; specifically, increased external mechanical tension is shown to induce 50% more mesenchymal‐to‐epithelial transition in heart progenitor cells, giving rise to cases of cardiac edema.^[^ ^8^ ^]^ Another study has shown that lack of tissue strain during gastrulation disrupts planar‐cell‐polarity in the ciliated epithelium of *Xenopus* embryos; importantly, this defect is rescued by applying exogenous strain similar to the normal gastrulation strain.^[^ ^21^ ^]^ Understanding how mechanics in general, and mechanical strain in particular, function in developmental processes is key to identifying underlying causes of diseases and birth defects.

High strain can also change tissue mechanical properties by fluidizing or hardening tissues, facilitating transitions between so‐called solid‐like and fluid‐like states by “unjamming” or “jamming” cells in the tissue.^[^ ^22^, ^23^, ^24^, ^25^ ^]^ Such transitions may involve alterations in adhesive junctional complexes between cells to allow remodeling.^[^ ^26^ ^]^ Recent studies have described “jamming” and “unjamming” tissue behaviors during embryonic development,^[^ ^22^, ^27^ ^]^ but it remains unknown whether or how mechanical strain alters cell–cell junctions enabling transitions between fluid‐ and solid‐like states. Nonetheless, the capacity of a tissue to remodel is critical for its ability to dissipate strain energy and the mechanical cues those strains encode.

Establishing causal relationships between mechanical cues and their effects on multicellular tissues requires tools capable of experimentally generating temporally and spatially defined strains, e.g., externally controlled strain rates, that are compatible with high‐resolution live cell imaging. High‐resolution live cell microscopy combined with image analysis pipelines can quantify the distribution of polarity factors, the dynamics of the cytoskeleton, adhesion, and membrane remodeling; and how those processes are coupled to signal transduction. The ability to control tissue strain over minutes to hours akin to controlling gene activity has immediate applications in studying the influence of mechanical cues in remodeling both synthetic and native tissues within complex micromechanical microenvironments.^[^ ^28^, ^29^, ^30^, ^31^, ^32^ ^]^

To test the physiological roles of strain, a tissue stretcher must be able to apply large strains to tissue samples cultured ex vivo as observed during embryonic morphogenesis. During the most rapid phases of embryonic morphogenesis, tissues experience large engineering strains, ranging from 50% to >100%.^[^ ^21^, ^33^ ^]^ For example, tissues undergoing convergent extension during zebrafish and *Xenopus* gastrulation and neurulation exhibit 1.5‐ to 2‐fold net changes in length, corresponding to 50% to 100% strain, with strain rates ranging from 10% to 20% per hour.^[^ ^1^, ^33^, ^34^, ^35^, ^36^ ^]^ Other phases of morphogenesis can exhibit higher strain rates, for instance, strain rates exceed 300% per hour during bottle cell respreading and archenteron elongation in *Xenopus* gastrulation (Unpublished, Lance Davidson).^[^ ^37^, ^38^ ^]^ Higher strain rates can be observed at the cell‐ or local tissue‐scale during cell division or wound healing.^[^ ^1^, ^39^ ^]^ To replicate these high in vivo levels of strain, we sought a stretcher system capable of 100% engineering strain. Furthermore, simultaneous observation of intracellular cytoskeletal and adhesion dynamics necessitates use of high numerical aperture oil immersion objective lenses that typically have small working distances, which requires samples to be within <200 µm from the coverslip. To acquire high‐resolution imaging sequences while applying strain, stable tissue mounts must minimize out‐of‐plane torsion that might drive samples out of the plane beyond the objective's working distance. Finally, the total mass of the stretcher device must be compatible with piezo or galvo‐driven z stages that are commonly used in rapid confocal sectioning in live cell imaging systems (e.g., Märzhäuser, and ASI).

Previously developed stretcher systems have been used to apply strain to live tissues in combination with microscopic analysis.^[^ ^40^ ^]^ Simple stretchers for suspended cell monolayers consist of wire cantilevers.^[^ ^41^, ^42^ ^]^ More sophisticated uniaxial or biaxial stretchers use clamps or posts to bond tissues to motorized actuators.^[^ ^43^, ^44^, ^45^ ^]^ Clamp‐ and actuator‐based systems are typically bulky and weigh considerably >250 g mass limit of fast z‐scanning stages. Another technique to apply strain is to induce compression along one axis and thus generate tensile strain along the other two axes.^[^ ^46^, ^47^ ^]^ Although this technique is easily implemented, it typically achieves only small strains and is limited to larger bulk tissue samples such as whole embryos. Indentation of an elastic substrate with seeded cells or tissues is also commonly used to induce tensile strain. In these indentation devices, an elastic substrate is fixed on posts, and an indenter is used to press on the substrate to deform the substrate, generating strain in seeded cells.^[^ ^48^ ^]^ Indentation requires steric access for positioning and travel along the *z*‐axis, which can also limit access for high‐resolution optics.

Past stretchers have also been designed to generate strain in cells or tissues by bonding or attaching them to an elastic substrate. The earliest efforts to stretch embryonic tissues used rubber substrates.^[^ ^49^ ^]^ Commercial systems (e.g., CellScale)^[^ ^50^ ^]^ use posts to fix the edges of an elastic substrate, with strain subsequently applied by a linear actuator along the edge of the device. Another commercial device (e.g., FLEXCELL),^[^ ^51^ ^]^ uses a macro‐scale indenter to induce strains up to 30%. None of these systems are well suited for high‐resolution confocal live cell microscopy.

To overcome the limitations of previous stretcher designs, we developed a stretcher, the TissueTractor, capable of inducing high strain in live tissues while simultaneously imaging at high‐resolution on an inverted confocal microscope. The TissueTractor has three main components: an easily interchangeable cassette, motorized actuators, and a custom microscope stage insert. The modular design of our stretching system enables integration with an inverted compound microscope equipped with high‐resolution confocal imaging. The cassette‐based design allows a simple exchange of samples for technical and biological replicates. Furthermore, the unique cassette design allows us to image samples directly through a standard #1.5 cover glass, instead of support substrates, such as an elastic substrate, which are not optimized for high‐resolution imaging. Additionally, the cassette design is easily modified to accommodate diverse cell types and tissues. In this study, we first demonstrate the use of the stretcher device with organotypic explants from *Xenopus laevis* embryos. Stretching tissues with the device allows us to quantify cell strain heterogeneity across the tissue and to visualize the remodeling of intracellular keratin filaments under tension. Next, we demonstrate the broader applicability of the system by modifying cassettes for use with human umbilical endothelial cells (HUVECs) and mouse neonatal cardiomyocytes, allowing observations of cell morphological changes under large strain. In conclusion, our innovative stretcher system enables high‐resolution confocal imaging of live tissues under high strain from diverse animal models. This flexible and customizable system is a powerful tool for gaining insight into how mechanical cues function in remodeling tissues.

## 2 Results

### 2.1 Design of a Customized Stretcher System for High‐resolution Live Imaging

**Figure 1:**
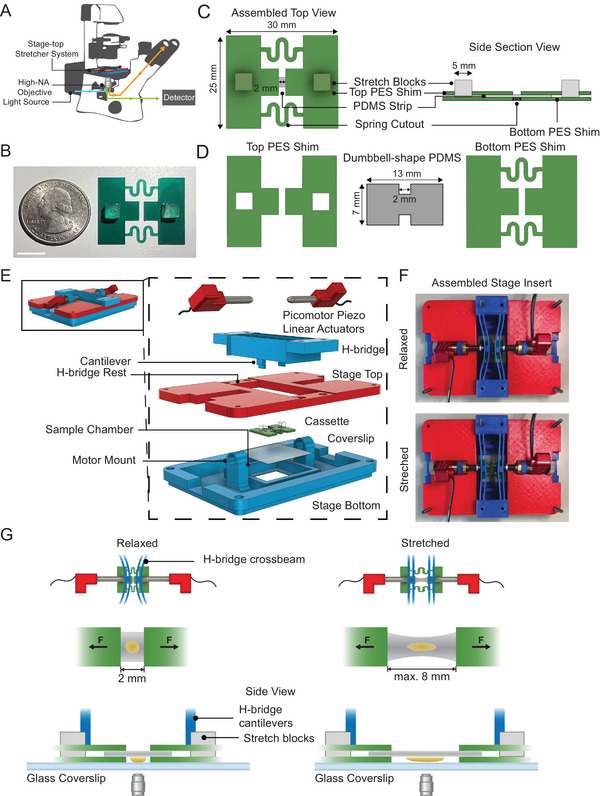
Configuration of the stretcher system. A) A schematic of the stage‐top stretcher system on an inverted microscope. B) A disposable cassette. Scale bar = 1 cm. C) Top and side section views of the assembled cassette. D) The cassette has a top PES shim with two square cut‐outs for stretcher blocks, a dumbbell‐shape PDMS substrate with a 2 mm exposed region after assembly, and a bottom PES shim to provide xyz‐stability. E) An exploded view of all components in the stretcher system. From top to bottom: two picomotor piezo linear actuators, an “H‐bridge” with extended cantilevers controlled by the movement of actuators, a stage top that has an H‐bridge rest to ensure a tight fit, a disposable cassette, and a stage bottom that has two motor mounts to mount the linear actuators, including a stage chamber with a glass coverslip bottom that can be filled with liquid. F) Top view of the assembled stage insert at a relaxed state (top) with H‐bridge crossbeams bending inward, and at a stretched state (bottom) with H‐bridge crossbeams straightened. G) A schematic of the cassette at a relaxed state with linear actuators pushing in to bend the H‐bridge crossbeams (top left); as the arms of the actuators retracted, H‐bridge crossbeams become straight and stretch the cassette (top right). A schematic of a tissue sample attached to the PDMS substrate of the cassette at a relaxed state (middle left) and at a maximum stretched state (middle right). A side view of a cassette being stretched and imaged on an inverted scope, with the tissue sample faces toward the objective lens (bottom).

To investigate how multicellular tissues respond to applied strain, we developed a microscope stage‐top stretcher integrated with commercial inverted brightfield and confocal microscopes to generate large effective mechanical strain in live samples (**Figure** **1**A; Figure S1, Supporting Information). The stretcher system features three main components: a cassette holding live samples, motorized linear actuators to deform the cassette, and a microscope stage insert to integrate actuators with the cassette (Figure 1B–F). The cassette provides stability along the *z*‐axis during preparation, handling, and stretching while allowing bilateral elastic deformation along the stretch axis (the *x*‐axis). The cassette is fabricated with a rigid thin polyester (PES) sheet using a 2D cutter. The cassette efficiently transmits tension to tissues attached to an elastomeric material, polydimethylsiloxane (PDMS), that spans a gap in the center of the cassette (Figure 1B–D). Tissues or cells are attached to the underside of the PDMS substrate for imaging with inverted microscopes, thereby avoiding imaging through the PDMS substrate and ensuring optimal optical clarity (Figure 1G). Since tissues and cell thickness can vary, we match the sample thickness with the thickness of the PES sheet used to build the cassette. We find PES sheets from 38 to 127 µm thick can accommodate the thickness of various samples so they remain within the working distance of a high numerical aperture objective lens. The stiff PES sheet provides a rigid mount for the two ends of the PDMS substrate. Samples attached to the substrate are physically coupled to the cassette; thus, as the cassette is stretched, samples are deformed. Our design uses 2D springs cut into the PES cassette. Spring designs were selected to minimize out‐of‐plane motion^[^ ^52^ ^]^ and to stabilize the tissue sample in the field of view during a stretch cycle. Additionally, the 2D springs enhance stability during assembly, cell culture, and handling of the cassette. Cassette rigidity and consistent bonding of the PDMS substrate to the cassette is achieved using a “sandwich” design, with a dumbbell‐shaped PDMS substrate bonded between the top and bottom PES sheets using UV‐curable optical adhesive (Figure 1C,D; Figure S2, Supporting Information). The dumbbell shape of the PDMS limits bond rupture and slippage from the PES at large strain (Figure 1D). Lastly, two 3D‐printed photopolymer resin block abutments mounted to the cassette allow direct contact between the two ends of the cassette and stage top apparatus (Figure 1C,D). A fully assembled cassette has a 2 mm wide gap, e.g., grip‐to‐grip (Figure 1C).

The custom, 3D‐printed microscope stage insert for the stretcher and cassette is designed to fit commercial inverted brightfield or confocal microscopes (Figure 1A; Figure S1, Supporting Information). To achieve an optimal combination of high‐resolution live imaging and effective mechanical manipulation of biological samples, the stage insert has four components: a stage insert bottom (specific to the microscope), a stage top, an “H‐bridge” cantilever, and two piezo linear actuators (Figure 1E,F). The stage insert bottom includes a sample chamber with a 45 × 50 mm glass coverslip and two motor mounts for securely mounting motorized linear actuators, providing stability, and preventing shifting during live imaging. The H‐bridge consists of two independent cantilevers with extensions to contact the inner surface of the two abutments on either side of the cassette (Figure 1D–G). The top of the H‐bridge aligns with the two linear actuators, so each motor can independently displace one cantilever (Figure 1G).

Stretch occurs as the two crossbeam cantilevers of the H‐bridge are relaxed, displacing the abutments on either side of the attached cassette. In preparation for uniaxial stretching, the two linear actuators are extended to deflect the crossbeams of the H‐bridge inward (Figure 1F,G). Next, the cassette is placed in a relaxed state in the culture‐media‐filled sample chamber, with the coverslip serving as the bottom of the chamber, allowing direct visualization of the sample (Figure 1G). The abutments of the cassette are aligned with, but not yet contacting, the lower extensions of the H‐bridges. At this point, the relaxed cassette is connected to H‐bridges by retracting the actuators. As the crossbeams of the H‐bridge relax, they contact the cassette abutments (Figure 1F,G; Video S1, Supporting Information). A small initial uniaxial bilateral stretch of the cassette immobilizes the sample within the field of view (Figure 1G). Motors, H‐bridge cantilevers, and the cassette with a PDMS substrate are rigidly integrated. Large‐scale strain is applied in steps as the cassette ends are moved apart and the PDMS stretches. The exposed PDMS substrate within the cassette can be stretched to a maximum of 8 mm, generating up to 300% engineering strain (grip‐to‐grip; from 2 to 8 mm) (Figure 1G). Cassette end movements are controlled either by computer‐controlled positioning of the linear actuators (LabView) or manually via a joystick. Image acquisition is carried out with microscope automation software (µManager 2.0).^[^ ^53^ ^]^ Stretch movements are in a stepwise motion and pause to allow imaging until the next movement command, allowing users to design experiments with distinct stretch movements and imaging intervals (Figure S3, Supporting Information).

Since large displacements can generate apparent drift of the image across the field of view, we developed custom software for microscope control, AutoCenter, to recenter the image. AutoCenter is based on the built‐in autofocus plugin of our microscope automation software (µManager 2.0).^[^ ^53^ ^]^ In brief, AutoCenter controls actuator movements and adjusts the position of the *xy* stage to counter the drift of the sample. The plugin module is executed at the beginning of each specified acquisition timepoint with two user inputs: 1) the channel to use for the adjustment, and 2) a search range along the z‐axis (usually equal to the z‐stack range set at the beginning of the acquisition). Using the user‐input search range, AutoCenter finds the z position that exhibits the greatest sharpness and captures a reference image. The module then calculates the *xy* displacement between the new reference image (from the current time point) and the previous reference image (from the last time point) using conjugate multiplication of the Fourier transforms of the two images, inverse transforming the result, and then finding the Δ*x* and Δ*y* deviations of the brightest pixel from the center of the image. Δ*x* and Δ*y* are used to set the stage to the new position in register with the earlier time point (Figure S4, and Video S2, Supporting Information).

### 2.2 Strain Performance of the Stretcher System

**Figure 2:**
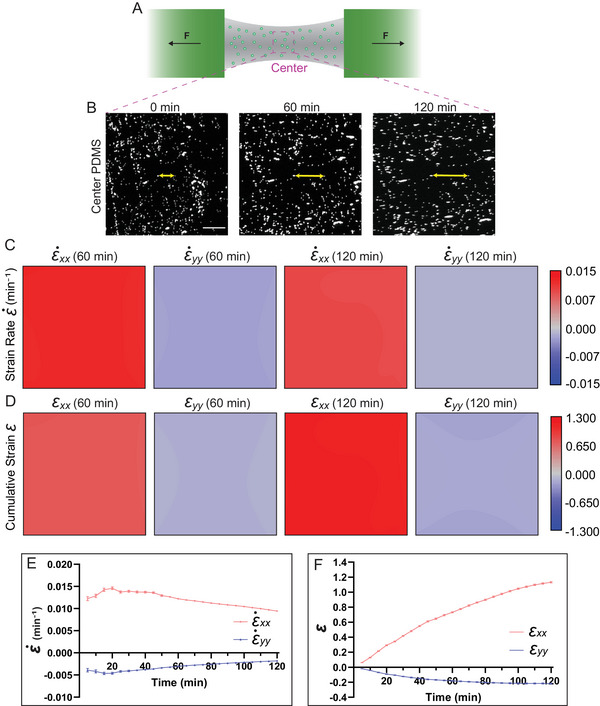
Characterization of the strain rate and strain profiles of the PDMS substrate. A) A schematic of the PDMS substrate coated with fluorescent beads. The Center (the magenta box) is indicated on the schematic. B) Representative fluorescent beads were traced at 0, 60, and 120 min; yellow arrows showing position changes between two beads. Scale bar = 200 µm. C) From left to right: ${\dot{\varepsilon }}_{xx}$ and ${\dot{\varepsilon }}_{yy}$ strain rate maps at 60 min, ${\dot{\varepsilon }}_{xx}$ and ${\dot{\varepsilon }}_{yy}$ strain rate maps at 120 min. D) From left to right: cumulative ε_*xx*_ and ε_*yy*_ strain maps at 60 min, cumulative ε_*xx*_ and ε_*yy*_ strain maps at 120 min. A phase lookup table is applied, where blue indicates contraction and red indicates elongation of the material. E) Quantifications of strain rates ${\dot{\varepsilon }}_{xx}$ and ${\dot{\varepsilon }}_{yy}$ at the center of the PDMS every 5 min during the stretch. F) Quantifications of cumulative strain ε_*xx*_ and ε_*yy*_ at the center of the PDMS every 5 min during the stretch. Error bars, standard deviation. *N =* 1 cassette. (Replicates, Figure S7, Supporting Information).

Substrate strain uniformity is critical in generating uniform deformation across attached samples. To validate strain uniformity, we coated PDMS in the cassette with 5 µm‐diameter green fluorescing polymer beads (**Figure** **2**A). The linear actuators were controlled by the LabView program to stretch the cassette at a constant velocity of 42.6 ± 4.11 µm per minute for 120 min (Figure S5, Supporting Information) and the cassette was imaged every 30 s (Video S3, Supporting Information). To verify the strain profile across the substrate, we focused on the performance of the center of the PDMS. We anticipated this region would have the most uniform strain and was the site planned for live tissue studies (Figure 2A). We tracked the fluorescent beads every 5 min during the stretch to investigate the strain uniformity at the center of the PDMS substrate; representative images of the fluorescent beads at the center of the PDMS substrate are shown for 0 (relaxed), 60, and 120 min (Figure 2B).

To quantify strain uniformity on the PDMS substrate, we used custom software for digital image correlation to analyze strain between image pairs across regions of interest (StrainMapper; Figure S6, Supporting Information).^[^ ^3^, ^54^, ^55^ ^]^ StrainMapper utilizes the B‐spline‐based plugin bUnwarpJ to quantify deformation between two images.^[^ ^56^ ^]^ bUnwarpJ assumes the image represents a continuous field and carries out a warping transformation that StrainMapper uses to calculate engineering strain (ε_*xx*_, ε_*yy*_, and ε_*xy*_) between a source image and a target image. StrainMapper outputs pixel‐by‐pixel maps of strain between image pairs sampled from the time‐lapse series (e.g., 0 to 5 min, 5 to 10 min, etc.). We defined the strain between consecutive images as “step strains” (**Table** **1**). Cumulative strains (e.g., 0 to 5 min, 0 to 10 min, and 0 to 60 min; Table 1) were computed by converting the series of engineering step strains, to true strain as the natural log of engineering step strain. Next, the cumulative true strain was calculated as the sum of the true strains over the frame pairs of the time series. Cumulative engineering strain was then recovered as the exponential of the cumulative true strain (See Experimental Section for details). Overall strain rates were then calculated by dividing the cumulative strain by the time elapsed in the time series images (Table 1). The difference between reporting strain at timed intervals and strain rate is not so critical as we validate stretching of the elastic PDMS substrate, however, in applying the TissueTractor to live tissues, strain rates are important to the biomechanics of live tissues and their mechanobiological responses.

**Table 1:** Glossary.

Terminology	Definition
Engineering Strain	The change of length (Δl = l‐l_0_) in a material in the direction of applied force is divided by the original length (l_0_) of the material. $\varepsilon =\frac{\Delta l}{l_{0}}\;$
Step Strain	The engineering strain between consecutive images sampled from the time‐lapse series (e.g., t_0_ to t_1_, t_1_ to t_2_, t_2_ to t_3_, etc.)
True Strain	The natural logarithm of the ratio of the final length of a material in the direction of applied force to its original length. $\varepsilon =ln(\frac{l}{l_{0}})$
Cumulative Strain	The engineering strain between the first image and the current image in the time‐lapse series (e.g., t_0_ to t_1_, t_0_ to t_2_, t_0_ to t_3_, etc.)
Strain rate [min^−1^]	Cumulative strain divided by time elapsed. $\dot{\varepsilon }=\frac{\varepsilon_{\mathrm{cum}}}{t}\;$

Strain rates across the center of the PDMS substrate at each time point were relatively homogeneous. For instance, strain rates ${\dot{\varepsilon }}_{xx}$ along the *x*‐axis (the stretch axis) and ${\dot{\varepsilon }}_{yy}$ along the *y*‐axis (perpendicular to the stretch axis) between 60 min and 120 min at the center of the PDMS substrate remained spatially uniform under applied uniaxial strain (Figure 2C). Elongation along the *x*‐axis in the given time interval resulted in a positive strain rate whereas shortening along the y‐axis in the given time interval produced a negative strain rate, (red and blue, respectively): ${\dot{\varepsilon }}_{xx}$(60 min) (1.2 × 10^−2^ ± 1.4 × 10^−4^ min^−1^), ${\dot{\varepsilon }}_{xx}$(120 min) (9.4 × 10^−3^ ± 8.4 × 10^−5^ min^−1^), and ${\dot{\varepsilon }}_{yy}$(60 min) (−3.0 × 10^−3^ ± 1.0 × 10^−4^ min^−1^), ${\dot{\varepsilon }}_{yy}$(120 min) (−1.8 × 10^−3^ ± 6.8 × 10^−5^ min^−1^) (Figure 2C,E). The colormaps at each time point indicated limited spatial variations in strain rates across the center region of the PDMS substrate (Figure 2C,E). Next, we investigated strain rate stability across time points. Strain rates ${\dot{\varepsilon }}_{xx}$ and ${\dot{\varepsilon }}_{yy}$ exhibited minor fluctuations, with a slight increase at early time points followed by a gradual decrease. The average strain rates were relatively constant with a standard deviation of 15% (${\dot{\varepsilon }}_{xx,Avg}$ = 1.2 × 10^−2^ ± 1.6 × 10^−3^ min^−1^ and ${\dot{\varepsilon }}_{yy,Avg}$ = −3.1 × 10^−3^ ± 9.1 × 10^−4^ min^−1^; Figure 2E). These small variations confirmed that strain rates were consistent across time points.

Having established the spatial and temporal uniformity of strain rates at the center of the PDMS substrate, we next considered the cumulative strains ε_*xx*_ and ε_*yy*_ to further assess the strain distribution and its behavior over the 120‐minute stretch. At 60 and 120 min, ε_*xx*_ reached 0.73 ± 8.7 × 10^−3^ and 1.1 ± 1.0 × 10^−2^, respectively, while ε_*yy*_ reached −0.19 ± 6.2 × 10^−3^ and −0.22 ± 8.2 × 10^−3^ (Figure 2D,F). Similar to strain rates, cumulative strains across the center of the PDMS displayed small variations, indicating a homogeneous strain distribution. However, we observed a nonlinearity in cumulative strains over the 120‐minute stretch (Figure 2F), likely due to the PDMS thinning at the grip sites (Figure S7A, Supporting Information). We suspected this was due to the dumbbell‐shape design of the PDMS and the fabrication process, in which the PDMS is coupled to the bottom PES shim without UV‐curable adhesive to minimize the distance between the sample and objective. However, the observed PDMS‐to‐grip bonding defect was consistent across cassettes, as strain profiles and strain rate profiles of different cassettes are similar (Figure S7B,C, Supporting Information).

Overall, the uniaxial strain rates ${\dot{\varepsilon }}_{xx}$, ${\dot{\varepsilon }}_{yy}$ and cumulative strains ε_*xx*_, ε_*yy*_ at the center of the PDMS substrate were homogeneous; thus, tissue samples attached to the center will experience consistently homogeneous substrate strain during stretching. For the remainder of this study, we focus on tissues and cells within the center of the PDMS substrate.

### 2.3 Tissue and Cellular Strains in *Xenopus laevis* Organotypic Explants

**Figure 3:**
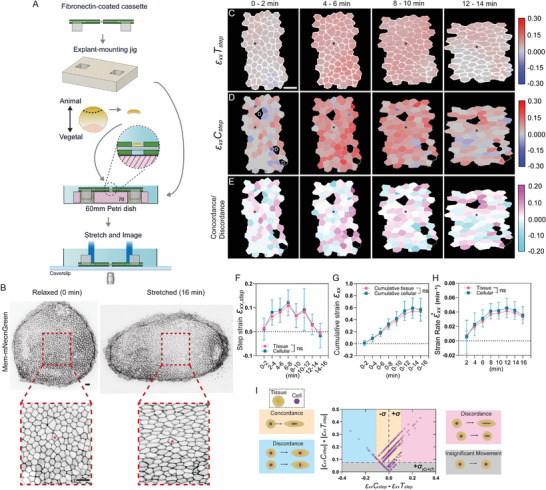
Tissue and cellular strains in *Xenopus laevis* organotypic explants. A) A schematic of mounting *Xenopus laevis* organotypic explant on the fibronectin‐coated cassette. An animal cap organotypic explant is microsurgically removed from the animal pole of a gastrula stage embryo attached to the PDMS substrate of the cassette and cultured to the desired stage. The cassette is flipped back and put into the microscope stage insert for stretching and imaging. B) A Stage 13 animal cap organotypic explant labeled with membrane‐mNeonGreen at relaxed state (0 min, left) and stretched state (16 min, right). The red dashed line outlines the region‐of‐interests, where the same region was traced and imaged through 8 stretch steps over 16 min in total. Scale bar = 35 µm. * indicates the same cell before and after stretching. C) ε_*xx*_ *T_step_* step strain map overlayed with cell outlines represents continuous tissue step strain over two consecutive stretch steps. D) ε_*xx*_ *C_step_* step strain mapped using individual cellular strain, which strain was calculated based on individual cell shape, with no variation of strain within the single cell. *N =* 81 cells. E) A concordance/discordance map is represented by the differences between ε_*xx*_ *C_step_* and ε_*xx*_ *T_step_*. A magenta‐cyan phase lookup table is applied, where both magenta and cyan indicate discordance behaviors between tissue and the individual cell, and white represents concordance. * indicates the same cell across the frames. “D” represents cells that divided throughout stretching. F) Quantification of both tissue and cellular step strains ε_*xx*, *step*_ between each two consecutive stretch steps. Error bars, standard deviation. n.s., not significant, *P* > 0.05, Multiple t‐tests were used as statistical analysis to compare tissue and cell step strains at each time point. G) Quantification of cumulative tissue and cellular strains ε_*xx*_ across the 8 stretch steps over 16 min in total. Error bars, standard deviation. n.s., not significant, *P* > 0.05, Multiple t‐tests were used as statistical analysis to compare tissue and cell cumulative strains at each time point. H) Quantification of both tissue and cellular strain rates ${\dot{\varepsilon }}_{xx}$ between each two consecutive stretch steps. Error bars, standard deviation. n.s. not significant, *P* > 0.05, Multiple t‐tests were used as statistical analysis to compare tissue and cell strain rates at each time point. I) Tissue‐cell concordance/discordance by plotting differences between ε_*xx*_ *C_step_* and ε_*xx*_ *T_step_* against absolute values of their sum. The grey region represents insignificant movement of both tissue and the cell. The yellow region represents tissue, and the cell strained concordantly. The schematic in the yellow box shows the concordant scenario when the tissue and the cell strain similarly. The magenta region represents discordance that the cell strained larger than the tissue. The schematic in the magenta box shows the discordant scenarios when the cell experiences a larger strain than the tissue. The cyan region represents discordance in that the tissue is strained larger than the cell. The schematic in the cyan box shows the discordant scenarios when the cell experiences a smaller strain than the tissue or even experiences a negative strain when the tissue experiences a positive strain. Tissue is represented by the tan oval; Cell is represented by the purple hexagon. σ_C + T_ = ±0.081, standard deviation of |ε_*xx*_ *C_step_* | + |ε_*xx*_ *T_step_* |. σ = ±0.057, standard deviation of ε_*xx*_ *C_step_* − ε_*xx*_ *T_step_*. *N =* 81 cells at 8 stretch steps (Replicates, Figure S9, Supporting Information).

To evaluate the stretcher system with live tissues, *Xenopus laevis* organotypic explants were stretched and imaged with an inverted brightfield microscope (Video S4, and Figure S8, Supporting Information). Then, to visualize cells with a confocal microscope, we injected membrane‐mNeonGreen encoding mRNAs into *Xenopus* fertilized eggs and cultured embryos to the early to mid‐gastrula stage. Regions of ectoderm expressing mNeonGreen were microsurgically dissected from the embryos. To observe apical cell surfaces during imaging, explants were cultured on the prospective undersurface of fibronectin‐coated PDMS bound in a cassette for at least an hour at room temperature. To attach explants to the bottom of the cassette, the fibronectin‐coated PDMS‐cassette was flipped and placed onto a 3D‐printed explant‐mounting jig, stabilizing the cassette horizontally for secure mounting of the explant to the PDMS undersurface (**Figure** **3**A, and Experimental Section). Once the tissue adhered to the PDMS, the cassette was inverted so that the apical face of the organotypic explant faced the objective, allowing tracking of the same group of cells during stretch (Figure 3B, red box: tissue, asterisks: cell).

To minimize image blurring that can occur during displacement, cell groups were tracked and imaged over 8 additive stretch steps, with a pause after each stretch step spanning 2 min to allow imaging (Figure 3B; Video s5, Supporting Information). In this case, each stretch step moved cassette grips apart by 375 µm, generating up to 150% strain (grip‐to‐grip) within the cassette; the initial relaxed state is indicated as 0 min, and the maximum stretched state, after 8 stretch steps, at 16 min (3 mm total grip‐to‐grip displacement). We selected the timespan between image pairs to minimize the impact of non‐linear inhomogeneous tissue behaviors such as cell division, intercalation, and rearrangement.

To compare tissue and individual cellular mechanical behaviors under tension, we used two independent analysis pipelines to quantify tissue strains and individual cellular strains along the *x*‐axis, roughly along the axis of stretch, and represented by the subscript *xx*. To closely observe how tissues and cells react under tension after each stretch step, we first used step strains (Table 1) between two consecutive stretch steps. As before, we applied custom image processing software (StrainMapper) to two confocal‐generated images to calculate tissue step strain ε_*xx*_ *T_step_* between two consecutive stretch steps. We found variations in step strains within the tissue in each stretch step (standard deviation, *σ* ≈ ±0.016) (Figure 3C,F) were greater than variations in the PDMS substrate step strain determined above (*σ* ≈ ±8 × 10^−4^). This suggests that the mechanical heterogeneity of the tissues under tension reflects sample variation rather than substrate heterogeneity. From this observation, we suspected that individual cells exhibit heterogenous strain. To measure strain on a cell‐by‐cell basis, we segmented and registered individual cells across stretch steps with segmentation software (Seedwater Segmenter).^[^ ^57^ ^]^ A custom image processing pipeline (FIJI and MATLAB)^[^ ^28^, ^58^ ^]^ was used to quantify shape and position information from segmented cells. From cell shape changes, we calculated the individual cellular step strain ε_*xx*_ *C_step_* between two stretch steps of cells that stayed in the imaging frame through all 8 stretch steps. The analysis excluded cells that divided during stretch, since those may not accurately reflect changes in strain that are solely due to the stretching. Similar to our findings from the bulk analysis pipeline, we observed heterogeneous cellular strain (σ varied from ±0.046 to ±0.088) across the tissue, with both positively and negatively strained cells (Figure 3D,F; Figure S9, Supporting Information).

We next wanted to investigate how consistently tissues and individual cells react to tension. Comparing ε_*xx*_ *T_step_* and ε_*xx*_ *C_step_* at each stretch step (0‐2 min, 2–4 min, etc.), we found no significant differences (Figure 3F). We also compared cumulative tissue and cellular strains ε_*xx*_ by calculating strain between the relaxed state (0 min) and each stretch step (2, 4 min, etc.). Again, we found no significant differences between cumulative tissue and cellular strains at each stretch step (Figure 3G), as well as strain rates (Figure 3H). To investigate cell‐to‐cell variation between tissue and cell strains, we mapped differences between the two analytic pipelines across the tissue reporting whether tissue and cell strains were in concordance or discordance (Figure 3E). To further investigate the concordance and discordance behaviors of tissues and cells, we adapted the concept of a *Z*‐score map^[^ ^59^ ^]^ by plotting the differences between cell and tissue step strains against their absolute sum to show the deviation of the differences between cell and tissue step strains from 0. A value of 0 indicates cell step strain and tissue step strain are the same. The concordance in cell and tissue strains are reported if the difference between tissue and cellular strains is within ±1 standard deviation from 0 (e.g., the cell stretches or compresses to a similar degree as the tissue; Figure 3I, yellow illustrations). If the difference is greater than ±1 standard deviation, the cellular and tissue strains are in discordance. We observed that 45% of tissue‐cell strains were concordant (Figure 3I, yellow region), suggesting consistent tissue and cell behaviors under tension. We also observed 1) 32% of tissues and cells only displayed small fluctuations in strain (Figure 3I, grey region), 2) 13% of individual cells strained more than the tissue, resulting in discordant strain (Figure 3I, magenta region), and 3) 10% of individual cell strains that were less than the tissue or even contracted while the tissue was stretched (Figure 3I, cyan region). Thus, we found that strain was heterogeneously distributed through *Xenopus* organotypic animal cap explants when under tension, with individual cells that showed either concordant or discordant strains within the tissue. Combining our novel stretcher with image analysis pipelines reveals a more complex tissue and cell response to strain.

### 2.4 High‐resolution Live Imaging of Various Model Systems on the TissueTractor

**Figure 4:**
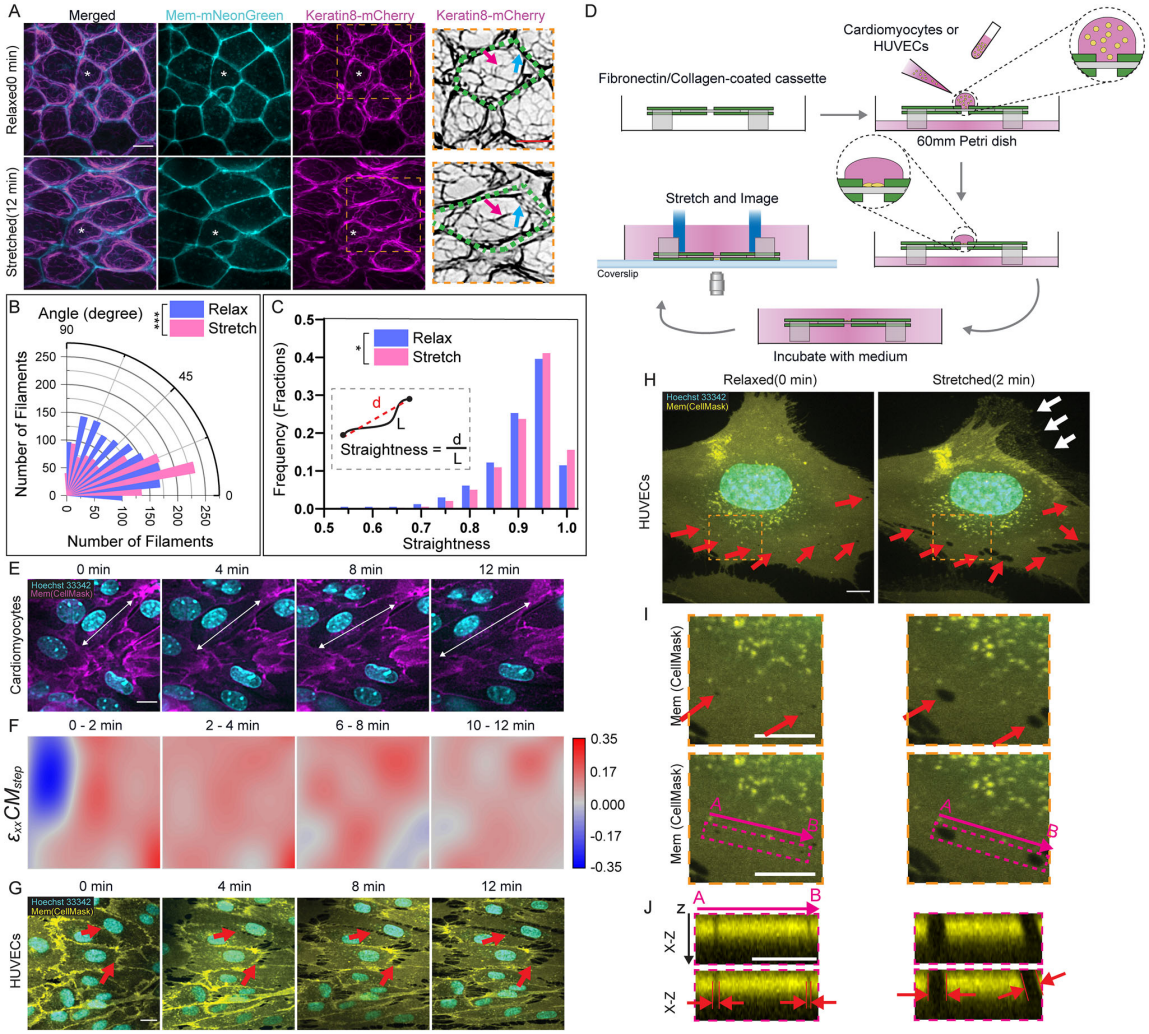
High‐resolution live imaging of various model systems on the TissueTractor. A) A Stage 11 animal cap organotypic explant labeled with mem‐mNeonGreen and keratin8‐mCherry at relaxed state (top) and stretched state (bottom). * indicates the same cell across frames. The last column shows keratin filaments in a single cell. The green dashed line outlines the single cell. The magenta arrow highlights tortuous filaments that straightened at the stretched state. The cyan arrow highlights the separation between the filaments and the cell junction. Scale bar = 10 µm. B) Filament orientation at the relax state (purple) and at the stretch state (pink). Keratin filaments were significantly more aligned with the stretch axis (0 degrees) after the stretch. *N =* 3 explants. The chi‐square test was used to compare distributions of filament orientations before and after stretch. *p <* 0.0001 ***. C) Filament straightness at the relax state (purple) and at the stretch state (pink). Straightness was calculated by dividing absolute distance d (red dashed line) by filament length L (black line). Fractions of filaments with straightness values <0.9 decreased while those with values more than 0.9 increased at the stretched state, indicating filaments straightened at the stretched state. *N =* 3 explants. A two‐tailed t‐test was used to compare filament straightness at relaxed and stretched states. *P* = 0.0141 *. D) A schematic of seeding cardiomyocytes or HUVECs on fibronectin‐ or collagen‐coated cassette. A droplet of suspended cardiomyocytes or HUVECs is pipetted onto the PDMS substrate of the cassette with the respective medium at the bottom of the petri dish. The cassette with cells at the desired state is flipped back and put into the microscope stage insert before imaging. E) Cardiomyocytes labeled with membrane and nucleus markers were stretched and imaged for 12 min over 6 stretch steps. White arrows indicate straining of the cell membrane. Scale bar = 10 µm. F) ε_*xx*_ *CM_step_* step strain map of cardiomyocytes during stretching. The contraction (shown in blue) was potentially due to the natural beating of cardiomyocytes. G) HUVECs labeled with membrane and nucleus markers were stretched and imaged for 12 min over 6 stretch steps. Red arrows highlight the tearing of cell–cell contacts. Scale bar = 20 µm. H) A single HUVEC at relaxed (left) and stretched (right) states. Red arrows highlight the fenestrated holes in the cell membrane. White arrows highlight the detachment of the cell membrane to the PDMS substrate. The orange dashed box outlines the zoomed‐in region shown in I. I) The zoomed‐in view of the cell membrane. Red arrows highlight the fenestrated holes before and after stretching. The magenta arrow and bar indicate the area corresponding to the *X–Z* view shown in J. The direction of the view is marked from A to B. J) *X–Z* view of two fenestrated holes before and after stretching. Red arrows indicate the sizes of the holes. Scale bar = 10 µm.

Building on the previous demonstration, we next sought to use the TissueTractor with high‐resolution confocal fluorescent live‐imaging, assessing the compatibility of the cassette with a high numerical‐aperture oil‐immersion objective lens. We stretched *Xenopus* organotypic explants that expressed an intermediate filament reporter, keratin8‐mCherry, and a membrane marker, membrane‐mNeonGreen. We were able to observe and track keratin filament changes at a single filament level using a high numerical‐aperture oil‐immersion objective lens (**Figure** **4**A). Prior to stretching, we observed many curved keratin filaments, suggesting a relaxed state. To quantify these changes, we measured the orientation of the keratin filaments at relaxed and stretched states. Filaments were significantly more aligned with the stretch axis (0 degrees) following stretch, indicating a directional reorganization in response to the applied strain (Figure 4B). Additionally, we evaluated the straightness of the filaments by dividing the absolute distance between their two tips by the filament length (i.e., a perfectly straight filament has a value of straightness of 1). After stretching, we observed that some filaments straightened slightly as stretch increased (Figure 4A, magenta arrows), with more filaments having straightness values >0.9, suggesting keratin filaments bear more load after stretching (Figure 4C). We also observed some cases where keratin filaments initially associated with the cell–cell junction would, at a stretched state, detach from junctions (Figure 4A, cyan arrows). These observations suggest that keratin filaments may act as key structural elements that redistribute mechanical load under tension, potentially through mechanisms involving filament elongation, realignment, or interactions with other components of the cytoskeleton.^[^ ^60^, ^61^ ^]^

We also tested two other live cell model systems in our stretcher system, mouse neonatal cardiomyocytes (CM) and human umbilical vein endothelial cells (HUVECs). To seed the cells on the cassette, as before, we prepared extracellular matrix (ECM)‐coated cassettes before the experiment by adsorbing either fibronectin (for HUVECs) or collagen I (for cardiomyocytes) onto the PDMS substrates. After coating, we inverted the cassette and added to the PDMS 30 µL of cell culture medium containing either cardiomyocytes or HUVECs. We incubated cells on the cassette until they were attached to the PDMS surface. Vital dyes for nuclei and plasma membrane were added to the medium 30 min before imaging. As with tissue explants, we inverted the cassette and placed it in the stretcher (Figure 4D, and Experimental Section). We applied strain and collected images over 6 stretch steps in 12 min with a 2 min pause between two consecutive stretch steps.

Cardiomyocytes appeared to maintain cell–cell contact while nuclear shape remained relatively unchanged during stretching (Figure 4E). Imaging the same population of cells throughout the stretch steps allowed us to map tissue step strain between each consecutive stretch steps ε_*xx*_ *CM* using StrainMapper (Figure 4F). Step strain heterogeneity was observed in cardiomyocytes across the field during stretching (Figure 4F), potentially due to their natural beating on the cassette, which causes cycles of expansion and contraction. Additionally, local membrane tearing was observed in the cardiomyocytes during stretching, leading to areas of negative strains (Video S6, yellow arrow, Supporting Information). Despite these effects, most cardiomyocytes under high strain remained connected to each other and continued to beat (Video S6, Supporting Information). This ability to withstand mechanical strain while maintaining functionality highlights the potential role of stretch‐induced biomechanical signals in promoting the structural and functional maturation of cardiomyocytes.^[^ ^62^, ^63^ ^]^

Since HUVECs are larger than Xenopus embryonic cells or cardiomyocytes, we initially used a 25× objective to image confluent layers to include more cells in the imaging frame. In contrast to our *Xenopus* embryonic epithelium and mouse cardiomyocytes, applied strain disrupted HUVECs cell–cell adhesions, and caused cell membranes to detach from the PDMS substrate (Figure 4G). Since stretching caused HUVEC membranes to retract and expose gaps between cells, we were not able to perform a tissue strain analysis. Next, we used a higher numerical aperture objective and observed single isolated cells under high substrate strain. We lowered the seeding density, allowing us to strain single HUVECs (Figure 4H). While not obvious at rest, single cells under high strain exhibited multiple holes (Figure 4H,I, red arrows), similar to fenestrations that have been described previously in other endothelial tissues.^[^ ^64^ ^]^ An *X–Z* side view indicated these holes penetrated through the thickness of the cell (Figure 4J). At increased strains, cell membranes appeared to rupture or tear where fenestrated holes had appeared at lower strains (Figure 4H–J, red arrows). Furthermore, cell‐substrate adhesion was also disrupted after stretching, leaving only streaks of membrane attached to the substrate (Figure 4H, white arrows). Comparing these two cell types, our preliminary observations indicate cardiomyocytes can remain attached to the substrate and maintain cell–cell contacts under strain. By contrast, highly strained confluent layers of HUVECs lost cell–cell adhesions, single cells ruptured at sites where fenestrations were observed, and both single and confluent HUVECs lost cell‐substrate adhesions.

## 3 Conclusion and Discussion

In this work, we developed a stretcher system, the TissueTractor, that enables effective mechanical stimulation of live samples while achieving high‐resolution live imaging. The stretcher makes use of an affordable 2D cutter and 3D printers to fabricate parts that can be integrated with low‐cost linear actuators. Key to the stretcher system is an exchangeable 2D‐cut cassette that keeps the sample within the working distance of a high numerical‐aperture objective lens. The cassette and two linear actuators are assembled into a custom‐designed microscope stage insert for simultaneous mechanical manipulation and visualization. The TissueTractor is designed to be compatible with both long‐working distance stereo microscopes, and inverted compound microscopes together with a range of objective lenses with high numerical aperture, such as 63× or 100× oil‐immersion objectives. To correct *xy*‐drift during imaging, we implemented a customized AutoCenter plugin for µManager.^[^ ^53^ ^]^ The TissueTractor can generate up to 300% (grip‐to‐grip) engineering strain within the cassette, resulting in over 100% strain in the PDMS substrate.

With the stretcher system, we were able to acquire high‐resolution live images of organotypic explants during stretching and analyze tissue and cellular scale engineering strain. In addition, we were able to visualize intracellular intermediate filaments under strain at a single filament level that were previously challenging to capture. This level of detail can provide a new understanding of how cells and tissues dissipate mechanical forces and maintain structural integrity under high strain. When testing cardiomyocytes from neonatal mice and HUVECs, we observed that cardiomyocytes retained cell–cell contacts, and nuclear shape, and continued beating under high strain, showing robust adhesion and strong junctional integrity. By contrast, HUVECs exhibited disrupted cell–cell adhesions, membrane detachment, and, in isolated cells, fenestration‐like holes that ruptured at increased strain levels, coinciding with adhesion loss. These findings suggest that cardiomyocytes are more resilient to mechanical strain, while HUVECs are more susceptible to adhesion and membrane disruption. These model tissues demonstrate the utility of the exchangeable cassettes and our stretcher system for visualizing and measuring the effects of strain in diverse cell and tissue types with high‐resolution confocal imaging. Although we only tested two animal models and one human cell type in this work, the stretcher system is compatible with most cultured cells, and organotypic explants that adhere to extracellular matrix.

The choice of extracellular matrix coating can influence cell signaling pathways and impact the cellular response to strain, depending on the cell type.^[^ ^27^, ^65^, ^66^ ^]^ For our experiments with *Xenopus* animal cap ectoderm, fibronectin—present natively within the embryo—was used as a coating, as it should not alter the tissue's response to mechanical strain. However, different ECM coatings may be required depending on cell type, as ECM composition can direct changes in cell behavior, including cell type, fate, and maturation.^[^ ^27^, ^65^, ^66^ ^]^ For instance, cardiomyocytes exhibit enhanced proliferation, reduced hypertrophy, and improved alignment and maturation when cultured on a heterogeneous ECM mixture compared to simpler substrates like fibronectin or gelatin alone.^[^ ^67^ ^]^ These findings suggest a future direction for our stretcher system, where customized ECM coatings could be explored to study how specific ECM compositions influence mechanotransduction and cell fate under strain across diverse tissue types.

While the TissueTractor offers effective mechanical stimulation and high‐resolution imaging capabilities, it has certain limitations. Primarily, it is designed for uniaxial stretching, limiting its applicability for studies requiring multi‐axial strain profiles. Additionally, the device is compatible exclusively with inverted microscopes, which may restrict its use in setups where upright microscopy is preferred. The device relies on a substrate for sample attachment, meaning cells are not freely suspended, which could impact cellular responses in models that require a more physiologically suspended environment. Manual assembly of the cassette introduces a risk of defects, such as substrate detachment, which necessitates inspection prior to use. Furthermore, since the substrate is elastic, greater thinning occurs at larger strains, which can lead to substrate slippage from the plastic shims. Finally, the sample size is constrained by the cassette dimensions, which limits the number of tissues or cells that can be examined. These limitations suggest areas for potential improvements in future designs to broaden the TissueTractor's application range.

Various improvements can be integrated to broaden the applications of the stretcher system. The cassette can be adapted to use other biocompatible adhesives to attach tissue samples to PDMS substrates such as cyanoacrylate, Cell‐Tak (Corning), or poly‐l‐lysine.^[^ ^5^, ^68^ ^]^ Furthermore, in the future we envision modifying the cassette design by changing grip positions or spring connections to provide different strain profiles (i.e., shear strain, biaxial strain) (Figure S10, Supporting Information), to mimic other dynamically changing microenvironments. Furthermore, the size of the cassette substrate can be increased to include larger numbers of tissue/cell samples for fixation. While the current stretcher design sought to track and visualize live cellular and intracellular dynamics during stretching, other systems with multiple coupled stretchers may achieve high‐throughput analysis including genomic or proteomic analysis of mechano‐responses.

Our stretcher system will enable the testing of putative mechanosensors and mechanotransducers in living cells and provide insights into the signaling pathways and gene regulatory networks that respond to mechanical stimulation. In particular, the ability to generate and sustain high strain with simultaneous high‐resolution confocal imaging makes this a unique tool to investigate the plastic behaviors of growing multicellular tissues and how mechanical cues and cell biology play a role in development and disease.

## 4 Experimental Section

### Preparation and Fabrication of the Stretcher System

The top and bottom microscope stage insert, and the H‐bridge were printed with Polylactic Acid (PLA; Prusa Research) filaments by a Fused Deposition Modeling 3D‐printer (Prusa i3 MK3S; Prusa Research). Polyester (PES) sheets were purchased from Precision Brand. Polydimethylsiloxane (PDMS) sheets (0.005′’ (127 µm), 40D, Gloss finish) were purchased from Specialty Manufacturing, inc. The top and bottom PES sheets of the cassette and the dumbbell‐shaped PDMS sheets were cut using a Roland CAMM‐1 GS‐24 Vinyl Cutter (Roland DGA) with a 25‐degree/.125 offset blade (USA‐C125; Roland DGA) to provide clean, smooth cutting edges. Cut PES and PDMS sheets were washed with 100% acetone, followed by an extensive rinse of 100% ethanol and double‐deionized water for 24 h. Rinsed products were dried between wax paper to prevent dust accumulation. Stretcher abutment blocks were 3D‐printed using stereolithography (Form 2; Formlabs) with photocurable clear resin (Formlabs), followed by a 20 min 100% isopropanol wash (Form Wash; Formlabs) to remove excess resin, and then cured in UV‐light (405 nm) chamber (Form Cure; Formlabs) for 2 h at 60 °C.

The bottom PES sheets were placed in a 3D‐printed jig for assembly (Figure S2, Supporting Information). The dumbbell‐shape PDMS sheet was placed onto the bottom PES sheet with the dumbbell parts aligned with the grips. Then, the top sheets were placed onto the bottom sheet using the jig. A thin layer of UV‐curable optical adhesive (Norland Optical Adhesive 63; Edmund Optics) was applied between the top surface of the PDMS and the top PES sheet. Two stretcher blocks were bonded into the two cut‐out holes on the top sheets (Figure 1D; Figure S2, Supporting Information). Once assembled, the cassette was placed into a UV‐light (350nm) chamber to cure for 2 h. Cassettes were stored at room temperature before use and discarded after experiments.

### Fluorescent Bead Coating

Green fluorescent polymer microspheres (30 µL) (5.0 µm diameter; 1% solids; Duke Scientific Corp.) were diluted in 420 µL of double deionized water. A cassette was flipped (stretcher blocks at the bottom) and 100 µL of the diluted fluorescent beads solution was added to the PDMS part of the cassette. The cassette was air‐dried and covered with aluminum foil until the liquid evaporated.

### Microinjection of Xenopus laevis Embryos and Organotypic Explant Mounting

All *Xenopus laevis* work was approved by the Institutional Animal Care and Use Committee (IACUC) and the University of Pittsburgh Division of Laboratory Animal Resources (Protocol #24014521). *Xenopus laevis* embryos were obtained by the standard procedure.^[^ ^69^ ^]^ 50 pg of membrane‐tagged mNeonGreen mRNA was microinjected into 4‐cell embryos. To visualize keratin 8 filaments, 100 pg of keratin8‐mCherry mRNA was microinjected into 4‐cell embryos. Injected and wildtype embryos were cultured in 1/3x modified Barth Solution (MBS)^[^ ^70^ ^]^ to desired stages.

Cassettes were flipped (stretcher blocks at the bottom) and put into an oxygen plasma cleaner (Harrick Plasma) for 2 min to activate the surface of the PDMS substrates. 0.025 µg µL^−1^ of fibronectin (Chem Cruz) was added onto the cassette immediately after plasma cleaning and incubated at room temperature for 1 hour. The cassette was then transferred into a 60 mm petri dish with a mounting jig in Danilchik's For Amy^[^ ^70^ ^]^ medium with antibiotic and antimycotic (Sigma) (Figure 3A). An organotypic animal cap explant was microsurgically removed^[^ ^39^ ^]^ at the early gastrula stage (Stage 10)^[^ ^71^ ^]^ and immediately transferred and positioned at the center of the PDMS substrate of the cassette (Figure 3A). A 1.5 mm × 12 mm glass coverslip bridge with high vacuum grease (DuPont) on both ends immobilized the explant. The cassettes with the explants were incubated at 14 °C or room temperature to the desired stage. The glass bridge was removed before the cassette was transferred to the stretcher.

### Human Umbilical Vein Endothelial Cell (HUVECs) Culture

Pooled human umbilical vein endothelial cells (HUVECs; Promocell) were cultured in a sterile humidified incubator in complete endothelial cell growth medium (EC Growth Medium 2/EGM2, containing 2% FBS; Promocell) and 1× antibiotic‐antimycotic (Thermo Fisher Scientific) at 37 °C under 5% CO2. Upon confluency, cells were rinsed with HEPES BSS (Detach Kit, Promocell) and treated with 0.04% trypsin/0.03% EDTA for 5–7 min at room temperature until cells detached. After adding trypsin neutralization solution, cells were centrifuged at 220 × g for 3 min and gently resuspended in fresh EGM2. The cells used here were maintained in EGM2 for a maximum of six passages.

### Mouse Neonatal Cardiomyocyte Isolation and Culture

All mouse work mouse work was approved by the Institutional Animal Care and Use Committee (IACUC) and the University of Pittsburgh Division of Laboratory Animal Resources (#22112123). Outbred Swiss Webster mice were used to generate cardiomyocytes for stretcher experiments. Neonatal mouse cardiomyocytes were isolated as described.^[^ ^72^ ^]^ Briefly, mouse pups were sacrificed at P2 and the hearts were removed, cleaned, minced, and digested overnight at 4 °C in 20 mm BDM (2,3‐butanedione monoxime) and 0.0125% trypsin in Hank's balanced salt solution. The next day, heart tissue was digested further in 15 mg mL^−1^ Collagenase/Dispase (Roche) in Leibovitz medium with 20 mm BDM to create a single‐cell suspension. Cells were pre‐plated for 1.5–2 h in plating medium (65% high glucose DMEM, 19% M‐199, 10% horse serum, 5% fetal bovine serum, and 1% penicillin‐streptomycin) to remove fibroblasts and endothelial cells. After pre‐plating, cardiomyocytes were counted manually on a hemocytometer, and cell density was adjusted to 3 000 000 cells mL^−1^ to seed onto the stretcher.

### HUVECs and Mouse Cardiomyocytes Seeding and Live‐labeling

Cassettes were flipped (stretcher abutment blocks at the bottom) and two 20 mm x 10 mm plasma‐blocking PDMS sheets were placed onto the PES sheet part of the cassette, exposing the PDMS substrate. This ensured that only the PDMS substrate was surface activated in the oxygen plasma cleaner (Harrick Plasma), and the PES sheet remained hydrophobic. The cassettes were surface‐activated in the oxygen plasma cleaner for 2 min. 0.025 µg µL^−1^ of fibronectin (Chem Cruz) or 0.25 µg µL^−1^ of rat tail Type I collagen (Millipore) were added onto the cassette immediately after plasma cleaning, followed by 1‐hour incubation at room temperature. The fibronectin‐coated cassettes were then stored at 4 °C until use. Collagen Type I was aspirated from the surface of the cassette, followed by 1 h UV curing. Collagen‐coated cassettes were washed using phosphate‐buffered saline (PBS; Sigma) and stored dry, covered with aluminum foil, at room temperature until use.

The plasma‐blocking PDMS sheets were removed from the cassette before seeding. 30 µL of HUVECs (250 000 cells mL^−1^) or mouse cardiomyocytes (3 000 000 cells mL^−1^) were placed onto the PDMS part of the fibronectin‐coated or collagen I‐coated cassette, respectively, to form a droplet on top of the PDMS substrate (Figure 4D).

Cassettes with HUVECs droplets were cultured at 37 °C for 2 h to allow cell attachment, and then 8 mL of complete growth medium (Endothelial Cell Basal Medium‐2 C‐22211, with Endothelial Cell Growth Medium 2 Supplement Pack C‐39211, Promocell) was added into the petri dish to fully submerge the cassette. Submerged cassettes were incubated for 16 h at 37 °C before imaging.

Cassettes with cardiomyocytes were cultured at 37 °C for 4 h to allow cell attachment. Due to the long incubation time, 500 µL of cardiomyocyte plating medium was added to the bottom of the petri dish to limit evaporation during incubation. After 4 h, 12 mL of cardiomyocyte plating medium was added to the dish to fully submerge the cassette. 16 h post‐plating, the plating medium was exchanged for cardiomyocyte maintenance medium (78% high glucose DMEM, 17% M‐199, 4% horse serum, 1% penicillin/streptomycin, 1 µm Ara‐C, and 1 µm isoproterenol) and incubated at 37 °C for 72 h before imaging.

To label cells for live cell imaging, cassettes were flipped back (stretcher abutment blocks at the top) and Hoechst 33342 (nuclear stain; 2 µg mL^−1^; Thermo Fisher Scientific) and CellMask Green (plasma membrane stain, 1x working solution; Thermo Fisher Scientific) were added to the medium. Cells were incubated at 37 °C in 5% CO_2_ for 30 min in stain prior to imaging.

### Stretcher System Setup and Imaging

The cassette was transferred and positioned at the center of the sample chamber of the stage bottom with 4 mL of respective medium. Then, the stage top was placed onto the stage bottom; two picomotor piezo linear actuators (8301NF; Newport) with fully extended arms were securely mounted at the motor mount. The H‐bridge was then inserted until the two ends fully contacted the H‐bridge rest of the stage top (Figure 1F). The assembled microscope stage insert was placed onto an inverted microscope (Figure S1, Supporting Information).

The picomotor piezo linear actuators can be controlled by a LabView program with customizable velocity settings or manually operated by a joystick (Figure S3, Supporting Information). Images at a relaxed state and at the end of each stretch step were acquired using an inverted compound microscope (Leica) with a 63x/1.40NA oil immersion or a 25×/0.95NA water immersion objective lens, equipped with a spinning disk scan head (Yokogawa) and a CMOS camera (Hamamatsu). Sequential images were acquired using microscope automation software (µManager 2.0).^[^ ^53^ ^]^

### Segmentation and Strain Analysis

PDMS deformation and tissue level strains were calculated using the custom ImageJ macro StrainMapper (Validation shown in Figure S6, Supporting Information). Image stacks were pre‐registered using an established ImageJ plugin StackReg^[^ ^73^ ^]^ selecting the Translation option. Specifically, StrainMapper first converted the transformations produced by bUnwarpJ^[^ ^56^ ^]^ between the source and target images into a raw pixel‐by‐pixel displacement file. It then used the *x*‐ and *y*‐displacements to calculate the engineering step strain ε_*xx*_, ε_*yy*_, and ε_*xy*_ (Table 1) at each pixel within a 3 × 3 pixel subarray for each image pair in a time series. Cumulative strain (Table 1) was computed by first converting the engineering step strain to true strain (Equation 1), summing the true strain values of each step (Equation 2), and finally converting the cumulative true strain back to cumulative engineering strain (Equation 3):

(1) $$\varepsilon_{i,true}=ln\left(1+\varepsilon_{i,eng}\right)$$

(2) $$\varepsilon_{cum,true}=\sum_{i=0}^{n}\varepsilon_{i,true}\;$$

(3) $$\varepsilon_{cum,eng}=e^{\varepsilon_{cum,true}}\;-1$$

To evaluate spatial variations in strain and strain rates in the PDMS substrate, values were extracted from 20 × 20‐pixel size boxes across each 400 × 400‐pixel image (i.e., 400 boxes in total for each condition).

Seedwater Segmenter^[^ ^57^ ^]^ was used to segment *Xenopus* epithelial cells. A custom FIJI macro was used to acquire cell ROIs and shape information from the segmented cells. A custom MATLAB m‐code calculated cell‐level engineering strain rates based on cell shape changes.^[^ ^58^ ^]^ Custom macros and codes are available in a public archive.

An established MATLAB‐based package CT‐FIRE^[^ ^74^, ^75^ ^]^ was used to segment and measure the orientation and straightness of keratin filaments in *Xenopus* epithelial cells before and after stretch. Minimum filament length was set to be 20 pixels.

### Statistical Analysis

All statistical analysis was performed in GraphPad Prism version 10.4.1. (GraphPad Software). Visualization of keratin‐8 filament orientation was performed in Origin 2025 (OriginLab). All data was represented as mean ± standard deviations. To compare tissue and cellular step strains, cumulative strains, and strain rates at each time point, multiple t‐test was performed. The Chi‐square test was used to compare distributions of filament orientations before and after stretch. A two‐tailed t‐test was used to compare filament straightness at relaxed and stretched states. The sample size of each experiment was listed in the figure legend. Statistical significance was found when *p <* 0.05.

### Ethical Statement

All *Xenopus laevis* and mouse work were approved by the Institutional Animal Care and Use Committee (IACUC) and the University of Pittsburgh Division of Laboratory Animal Resources (Protocol #24014521 and #22112123, respectively).

## Conflict of Interest

The authors declare no conflict of interest.

## Author Contributions

The study was conceptualized by J.Y., E.H., D.V., T.W. and L.D. with methodology developed by J.Y. and L.D. The software was implemented by J.Y., D.V., S.A. and L.D. while formal analysis was conducted by J.Y. and L.D. The investigation involved J.Y., Y.C., S.B. and Y.D. Resources were provided by J.Y., E.H., Y.W., D.V., C.S., A.K. and L.D. The original draft of the manuscript was written by J.Y. and L.D. with review and editing contributions from J.Y., E.H., Y.W., D.V., T.W., S.A., C.S., Y.C., S.B., Y.D., A.K. and L.D. Visualization was handled by J.Y. and the project was supervised by L.D. Funding acquisition was managed by A.K. and L.D.

## Supporting information



Supporting Information

Supplemental Video 1

Supplemental Video 2

Supplemental Video 3

Supplemental Video 4

Supplemental Video 5

Supplemental Video 6

## Data Availability

Source data are provided with this paper. All other data that support the findings of this study are available from the authors upon reasonable request. Custom macros and codes are available in a public archive.
