# Supplementary material for: The TissueTractor: A Device for Applying Large Strains to Tissues and Cells for Simultaneous High‐Resolution Live Cell Microscopy
Source: Small Methods. 2025 Mar 9;9(8):2500136. doi: 10.1002/smtd.202500136 (PMC12353401; doi:10.1002/smtd.202500136)
Supplement: Supplementary file 1 — Supporting Information [file SMTD-9-2500136-s005.docx]

Supporting Information

**The TissueTractor: a device for applying large strains to tissues and cells for simultaneous high-resolution live cell microscopy**

*Jing Yang, Emily Hearty, Yingli Wang, Deepthi S. Vijayraghavan, Timothy Walter, Sommer Anjum, Carsten Stuckenholz, Ya-Wen Cheng, Sahana Balasubramanian, Yicheng Dong, Adam V. Kwiatkowski, and Lance A. Davidson**

**
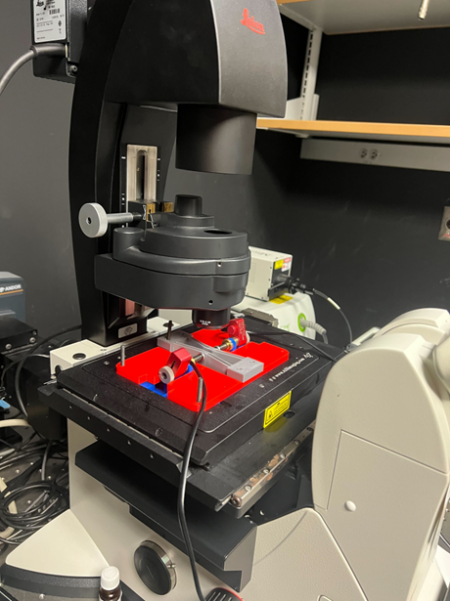

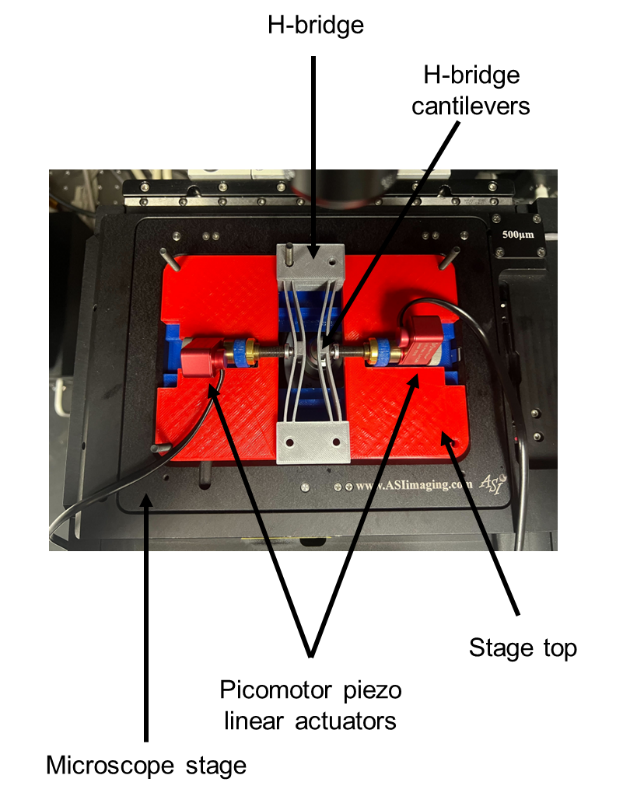
**

**A**

**B**

**Figure S1.** 3D-printed assembled microscope stage insert on a custom-built spinning disk confocal microscope. A) Top view of the stage insert on a microscope stage. The H-bridge sits on the stage top with crossbeams pushed inward by the picomotor piezo linear actuators that are mounted on the stage insert. B) a diagonal view of the microscope stage insert.


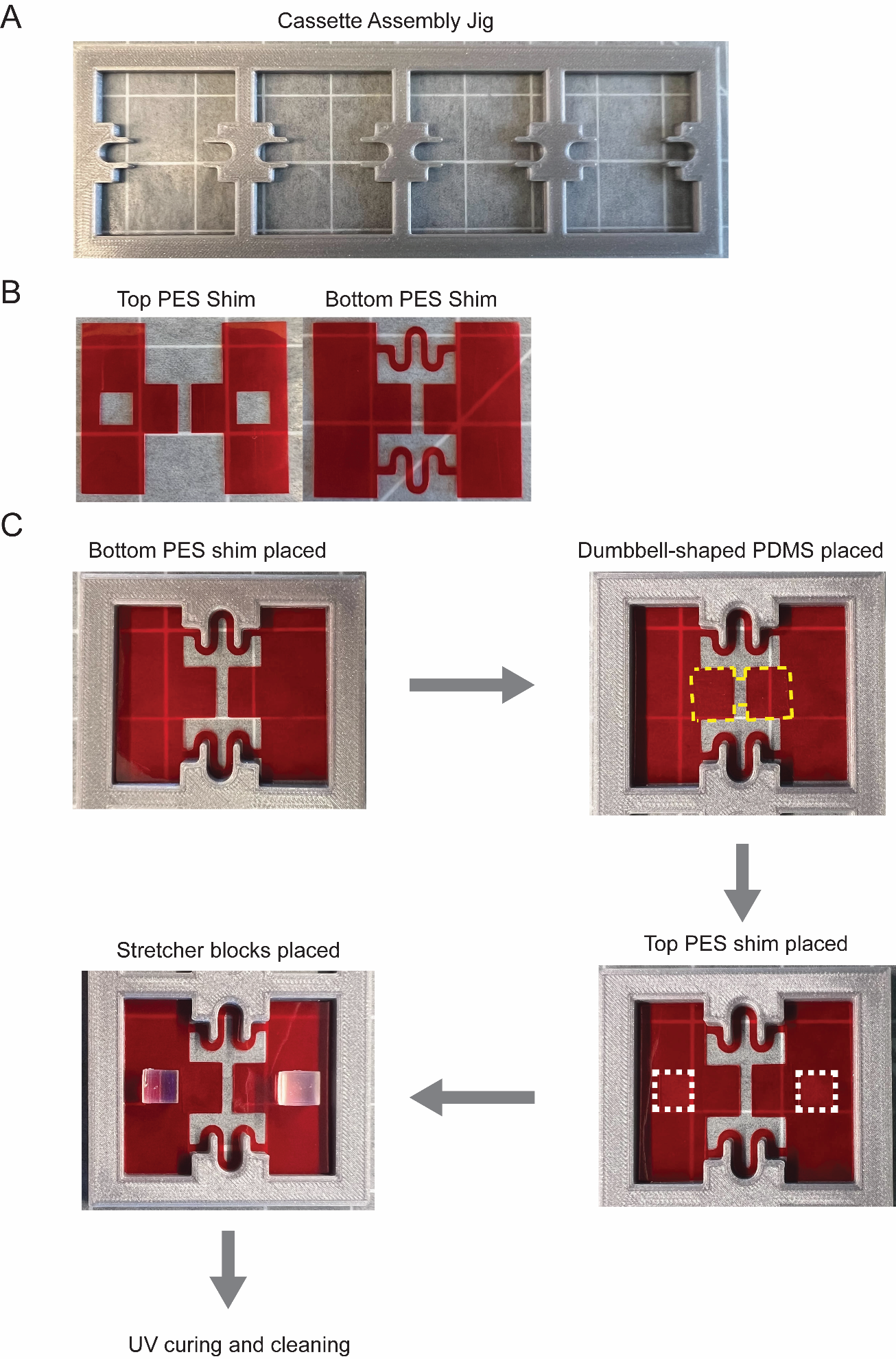


**Figure S2.** Cassette Assembly using the 3D-printed jig. A) an FDM-printed jig with four cassette-shape compartments. B) 2D-cut top PES shim with two cutouts for stretcher blocks alignment, and the bottom PES shim with spring cutouts. C) Assemble the cassette using the jig. The bottom PES shim was placed in one of the compartments; a dumbbell-shaped PDMS sheet was placed onto the middle part of the cassette (yellow dashed line); then the top PES shim was placed onto the cassette, followed by stretcher blocks with optical adhesive (white dashed boxes indicated the cutouts for stretcher blocks). Then the assembled cassette was cured in UV chamber and washed with 100% ethanol and double deionized water.

**
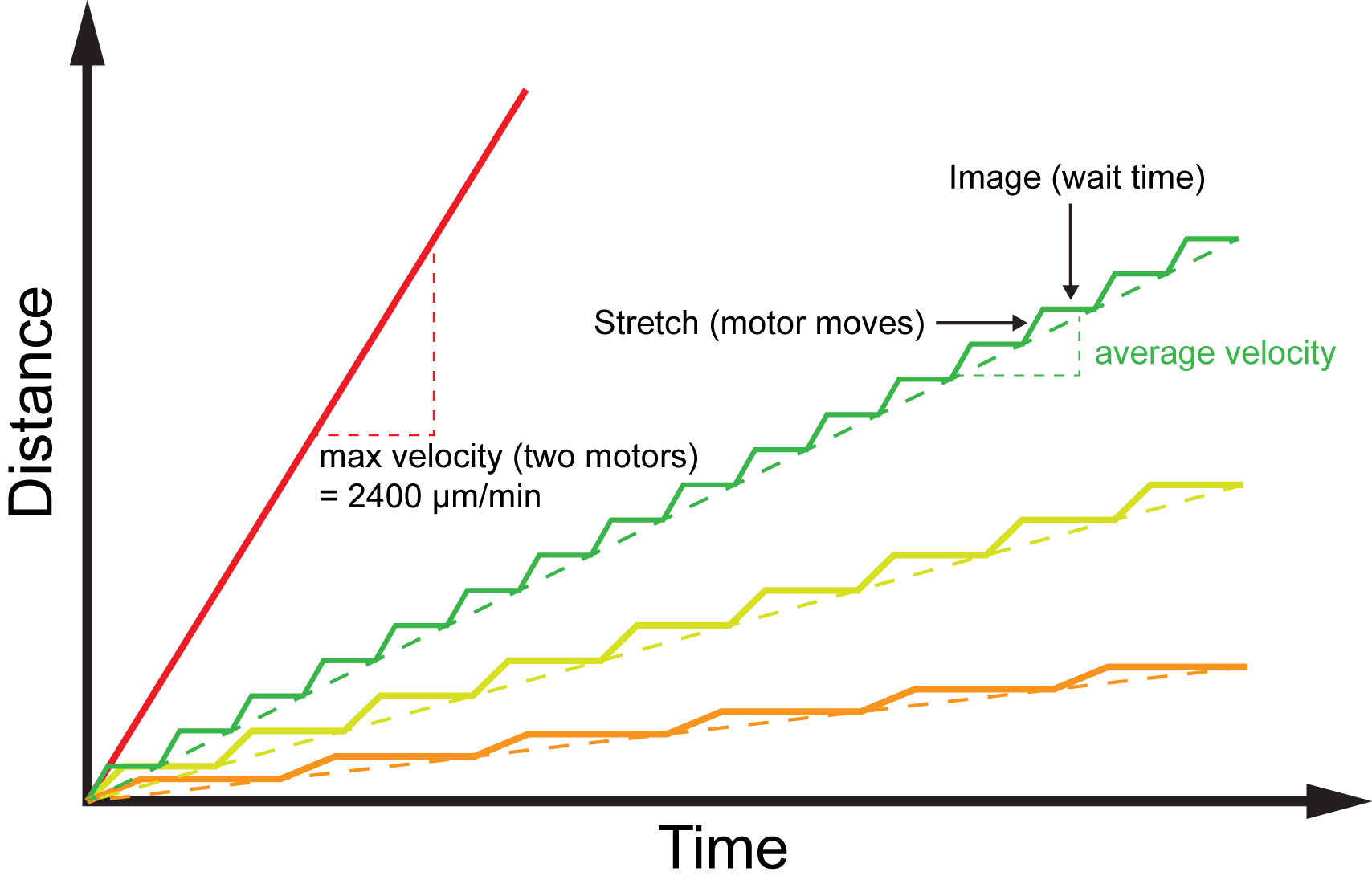
**

**Figure S3.** A schematic of estimated velocity profiles. After each stretch movement the motor pauses to allow imaging until the next movement command. A single piezo actuator used in the stretcher system is capable of achieving velocities ranging from 0.03 to 1200 μm/min (Newport 8301NF). The 30 nm resolution of the piezo-actuator enables precise position and velocity control and allows users to design experiments with distinct stretch movements and imaging intervals (estimated velocities, and the resulting strain rates, decrease from green to yellow to orange). The potential maximum velocity of the two motors combined is 2400 μm/min could result in 120%/min maximum strain rate (red line; PDMS strain reported grip-to-grip, from an initial gap of 2 mm and maximum grip-to-grip distance of 8 mm. Due to the control of the motor and the stepwise nature of our experimental design, there is technically no minimum velocity profile. For example, to achieve a very slow velocity, the motors can move in 60 nm increments with a 2-minute pause between stretches, resulting in an approximate velocity of 0.03 μm/min (1.8 μm/hour), which would be equivalent to 0.0015%/min strain rate.

**
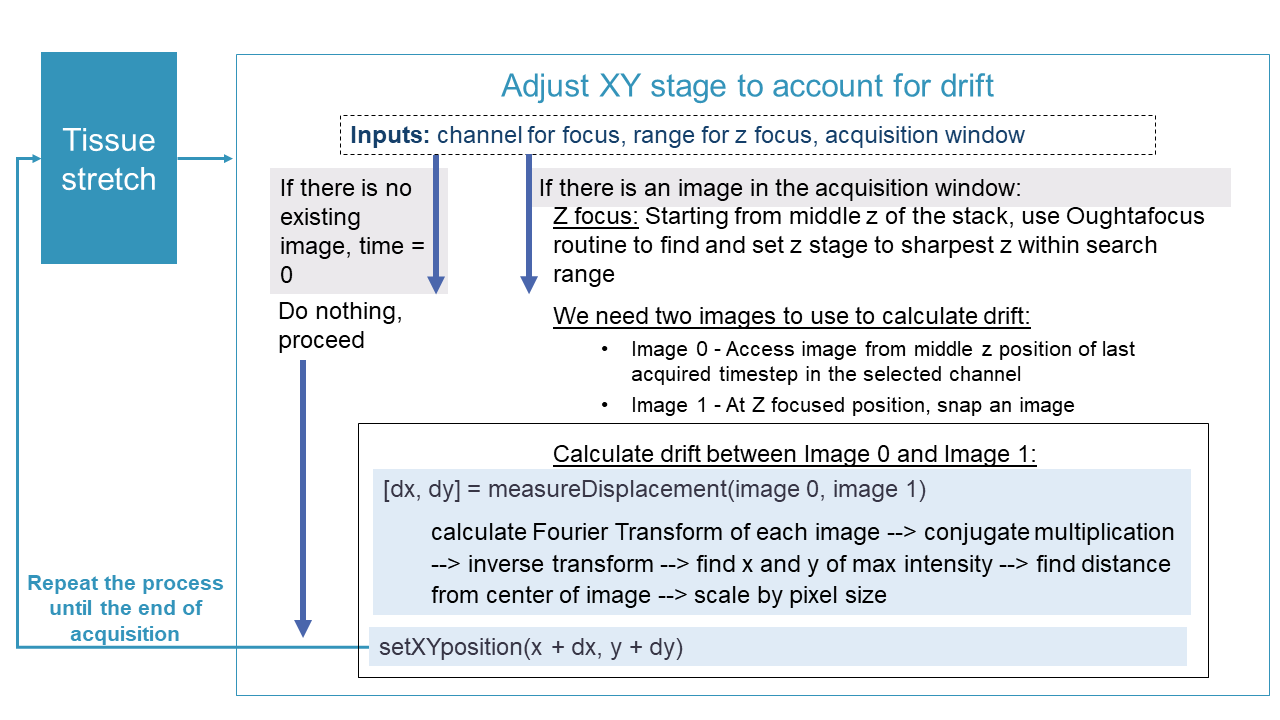
**

**Figure S4.** A flowchart for customized AutoCenter plugin. Between stretches of the motor, the xy stage is adjusted to account for drifting of the tissue that occurs during stretching. This is implemented with a custom autofocus plugin for Micro Manager, which handles image acquisition through ImageJ. This plugin uses the core autofocus functions, making it appear in the autofocus menu. The plugin module to adjust the xy stage to account for drift is executed at the beginning of each specified timepoint of image acquisition. The user supplies two inputs: the channel to focus in and the um range to use for z focus at the beginning of each timestep. First, the acquisition window is checked for an image. If no image exists, the plugin will do nothing and wait for the next round of images. If there is an image in the acquisition window, the Oughtafocus routine will be run from the middle of the stack plus and minus half the search range. This sharpest z is used to set the z position of the stage. Two images are needed to calculate the displacement. The first is taken from the middle z of the stack for the previous timepoint. The second is snapped at the newly focused z and stored within the plugin so it does not interfere with the acquisition. The displacement between these two images is calculated by conjugate multiplication of the Fourier Transforms of the images, inverse transforming the result, and then finding the deviation of the brightest pixel from the center of the image. This calculated dx and dy is then multiplied by the pixel size and used to set the new stage position.


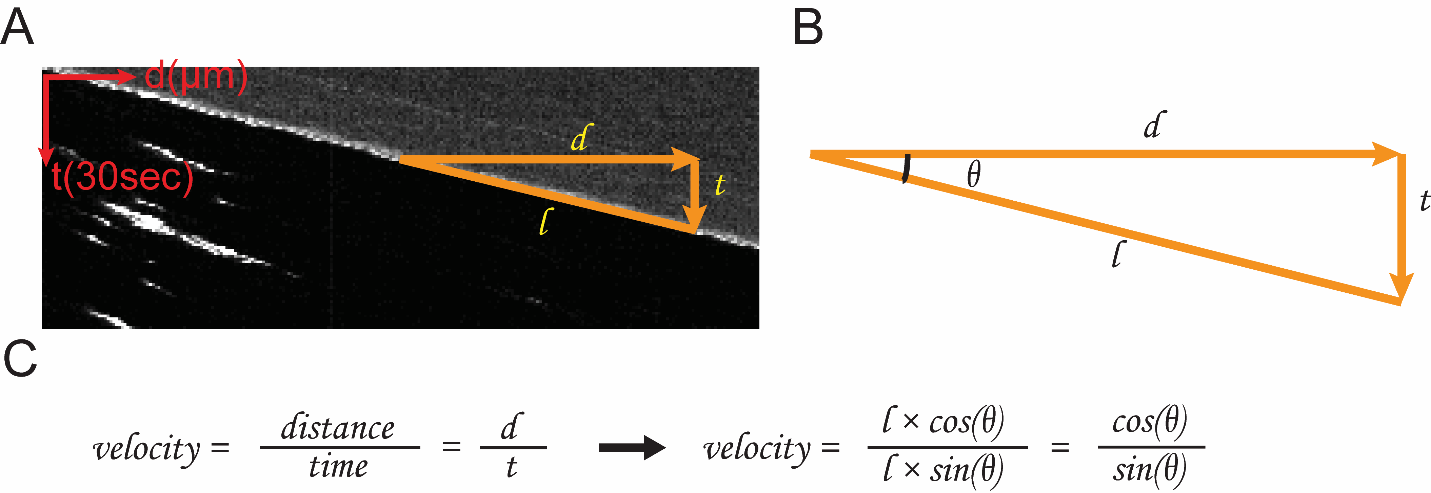


**Figure S5.** Calculate the velocity of the grip sites of the cassette. A) Reslice of the timelapse video of one of the cassette edges, showing a kymograph-like trajectory of the grip site traveled in distance (horizontal axis) and time (vertical axis). The edge appeared to travel in a straight line, indicating constant velocity during stretching. B) A right triangle with an angle *θ* was used to calculate the velocity, with the horizontal leg as the distance *d* and the vertical leg as the time *t*. The length of the hypotenuse was represented by $l$*.* C) Velocity of the one side of the cassette grip site was calculated by dividing distance by time. Velocity = 42.6 ± 4.11 µm/minute for two motors together, N = 10.


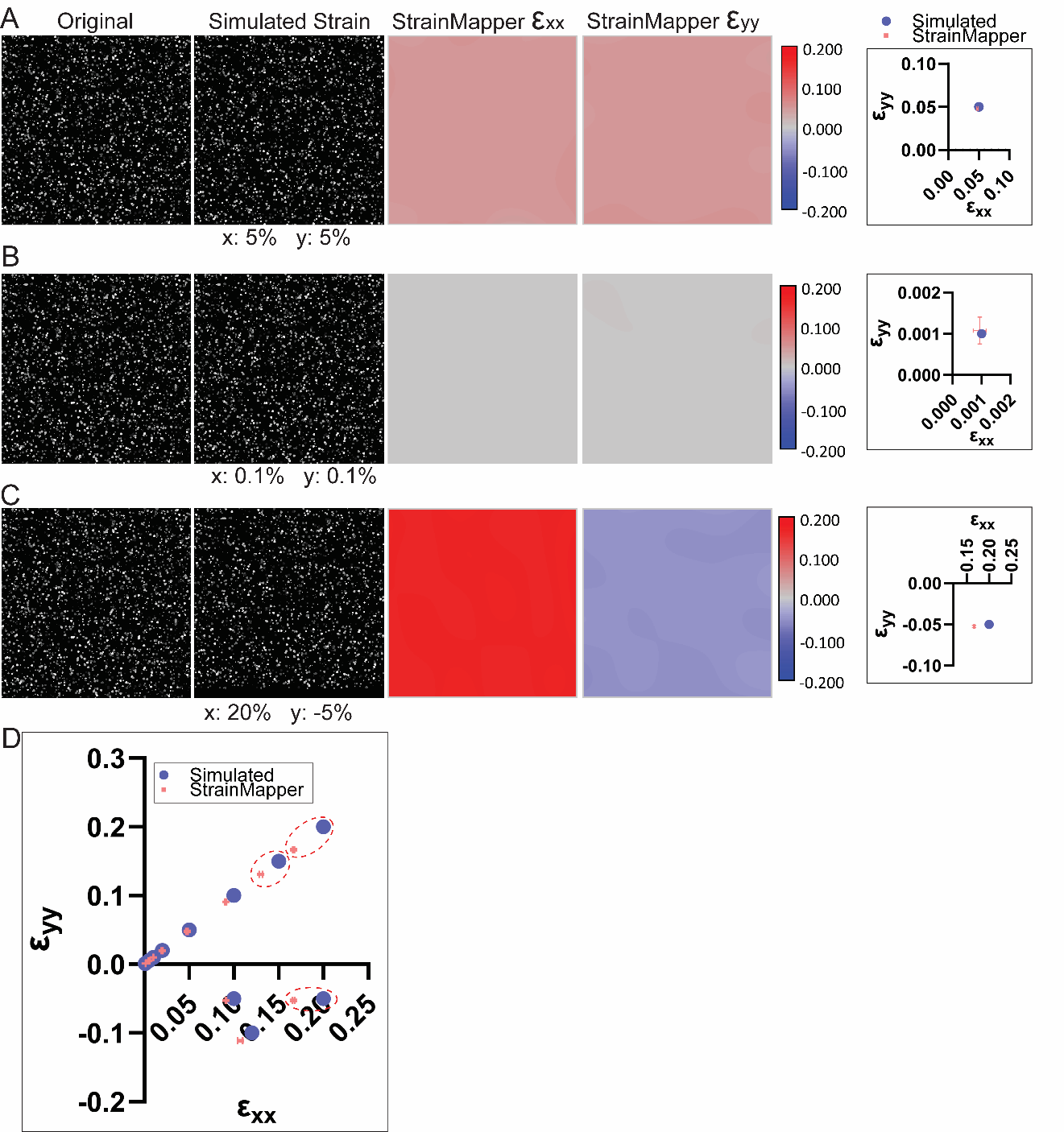


**Figure S6.** Validation of StrainMapper. Fiji is used to generate a pattern of random dots and simulate engineering strain along x- and y-axis on that pattern. StrainMapper is used to measure between the simulated image pairs. A, B, C) From left to right: original simulated random dots image, simulated strain from the original image, strain measured along x-axis, and y-axis, and plots of simulated strain and strain reported by StranMapper. a, simulated 5% $\varepsilon_{xx}$ and $\varepsilon_{yy}$; b, simulated 0.1% $\varepsilon_{xx}$ and $\varepsilon_{yy}$; c, simulated 20% $\varepsilon_{xx}$ and -5% $\varepsilon_{yy}$. D) 10 pairs of simulated and StrainMapper calculated strains. Dashed circles indicate cases where measured strains deviate from the simulated strain. Error bars, standard deviation.


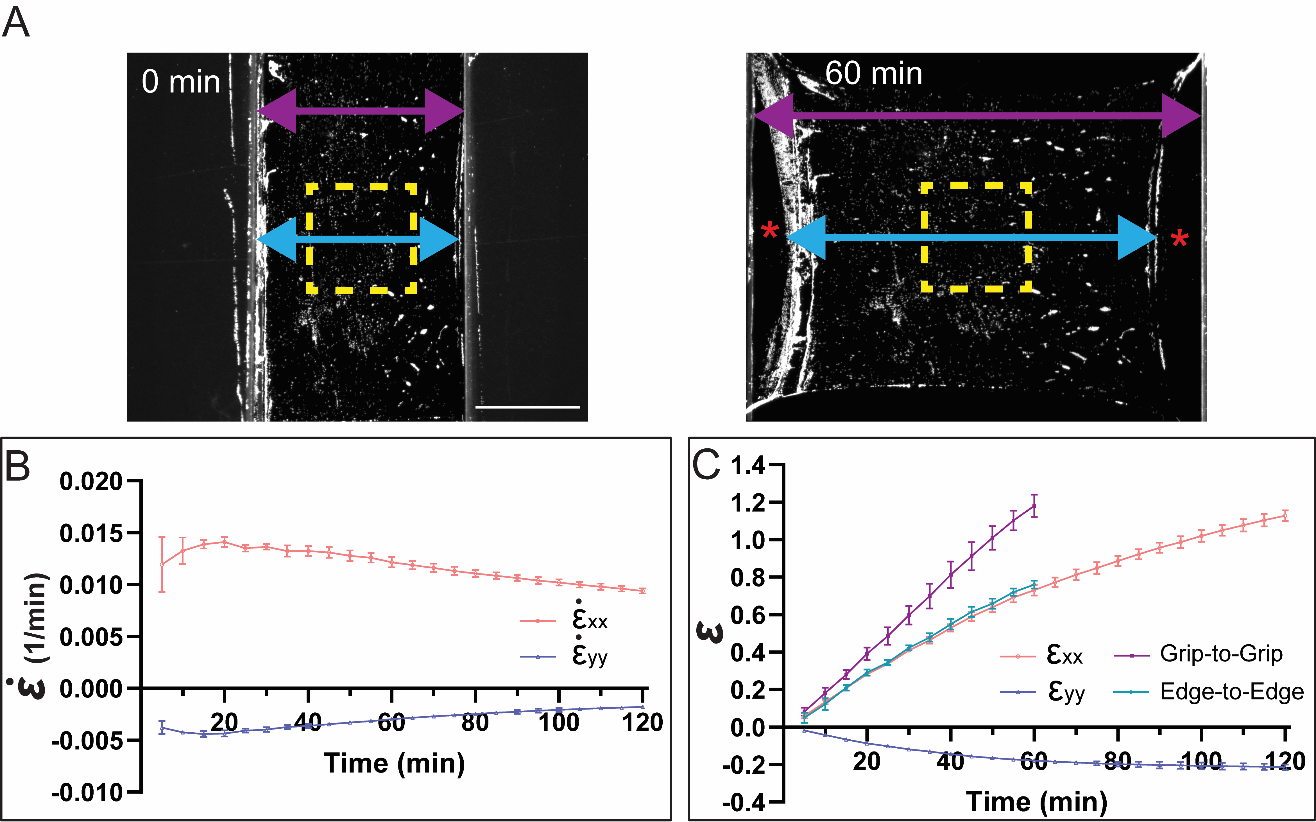


**Figure S7.** Strain and strain rate measurements of multiple cassettes. Variations among cassettes were considerably small in both strain and strain rate profiles. The nonlinearity of the axial strain at the center of the PDMS was also consistent across cassettes. A) Images of the grip-to-grip region of the cassette at relaxed (0 min) and stretched (60 min) states. Debonding of PDMS from the grip, e.g. edge of the PES shim (red asterisk), occurred during stretching. Purple line represents the distance between two grips; cyan represents the distance between the curved edges; yellow box represents the center of the PDMS substrate. The curving was consistent across cassettes. B) Strain rate $\dot{\varepsilon}_{xx}$ and $\dot{\varepsilon}_{yy}$ at the center of the PDMS substrate every 5 minutes during the 120-minute period (n = 3 cassettes). C) Cumulative strain $\varepsilon_{xx}$ and $\varepsilon_{yy}$ at the center of the PDMS every 5 minutes during the 120-minute stretch. Grip-to-Grip and Edge-to-Edge strains only calculated for the first 60 minutes. The grip-to-grip strain was relatively linear, indicating linear motion of the cassette grips. The minor debonding of the PDMS from the grips caused nonlinearity of the axial strain, which was the result of PDMS thinning at the grip sites caused by the dumbbell-shaped design and fabrication process. While the center strain of the PDMS did not match the grip-to-grip strain, the edge-to-edge strain was consistent with the center strain of the PDMS across cassettes, with minimal variations in strain and strain rate profiles, indicating the observed PDMS-to-grip bonding defect is consistent across cassettes. n = 3 cassettes. Error bars, standard deviation.


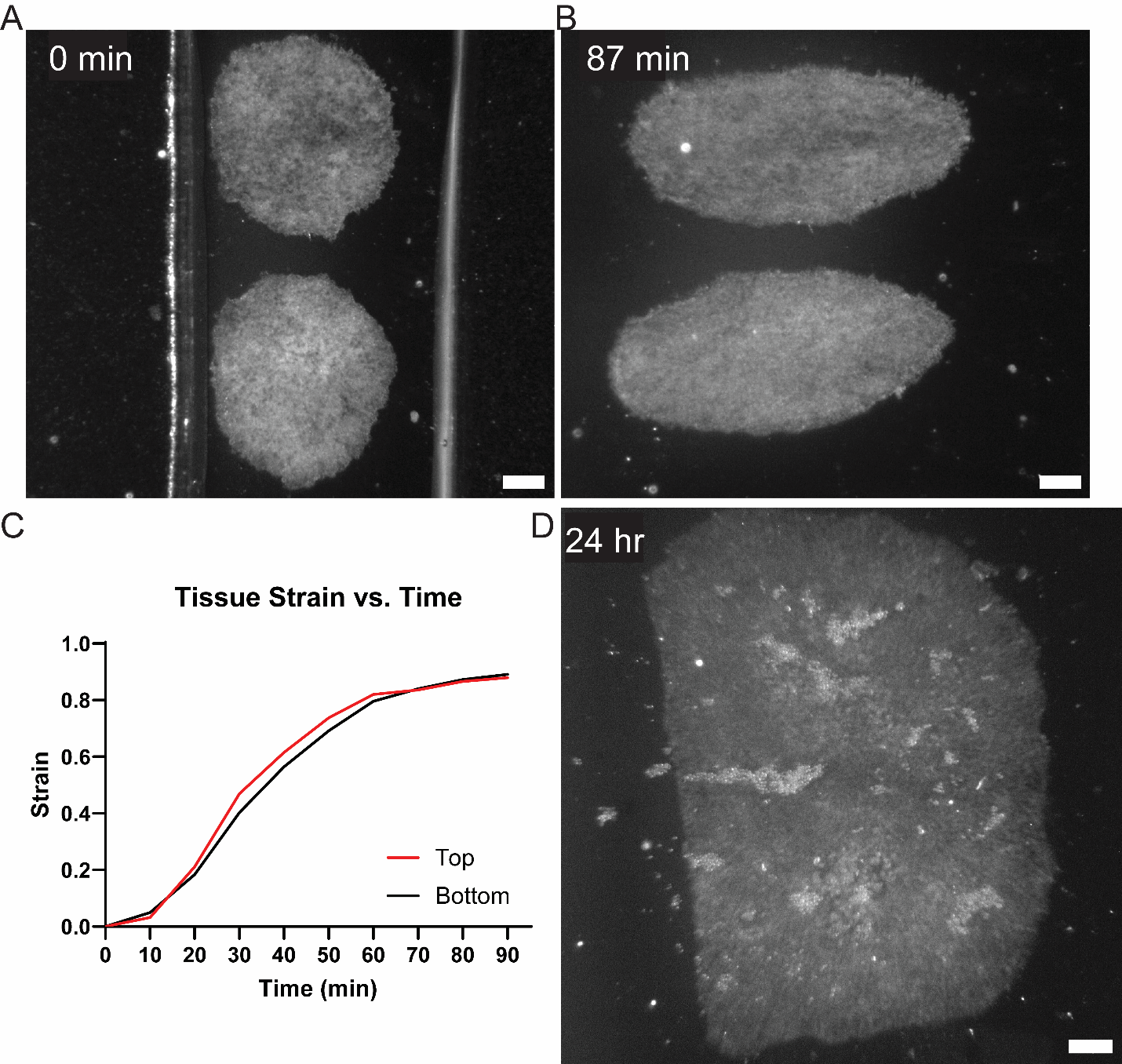


**Figure S8.** Two animal cap organotypic explants stretched and imaged with brightfield inverted microscope. A) at relaxed state (0 min); B) at stretched state (87 min). C) Quantification of engineering strain from top and bottom explants during the stretch. D) Growth and spreading of the two explants after 24 hours post stretch. Explants were held at the stretched state. Scale bar = 200 μm.


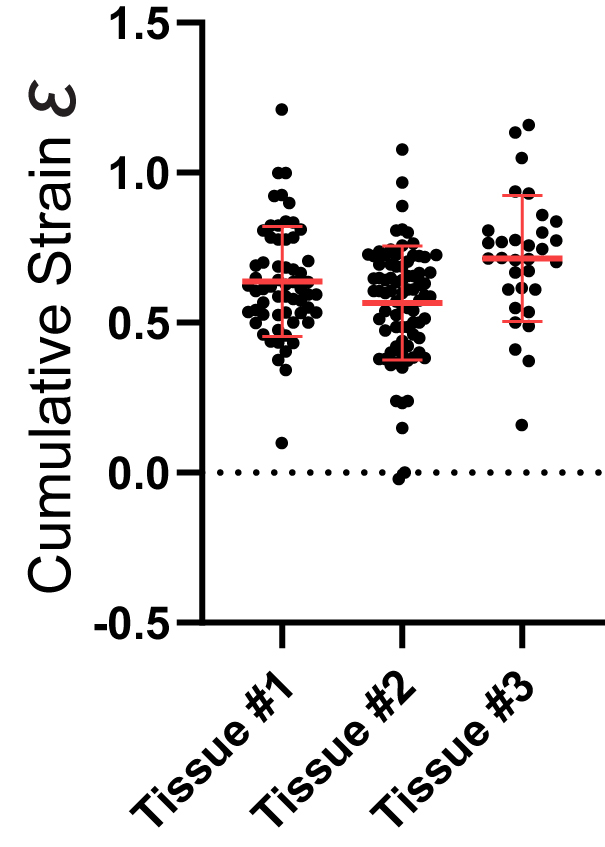


**Figure S9.** Cumulative strain of individual cells within the tissue sample at the end of the stretch (16 minutes). Cellular strains in all three tissue samples showed considerable variations (σ_1_ = 0.183, σ_2_ = 0.190, σ_3_ = 0.209). Cells were sampled from the central region of the organotypic explant, which were placed at the center of the PDMS substrate in the cassette. Error bars, standard deviations.


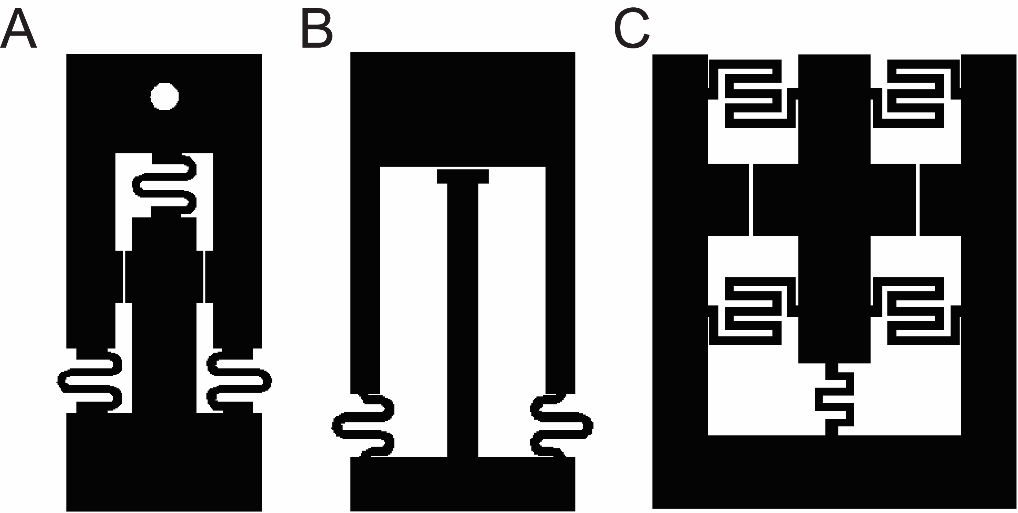


**Figure S10.** Cassette designs for different strain profiles. A) a design for pure shear strain; B) a design for unilateral stretch; C) a design for biaxial stretching and shearing. Red boxes indicate substrates attachment.

**Supplementary Video 1**

Video of the top and bottom views of the stretcher system during stretching process. The cassette was in a relaxed state with the crossbeams of the H-bridge bent inward. As the arms of the linear actuators retracted, crossbeams straightened and stretched the cassette.

**Supplementary Video 2**

A fixed convallaria microscope slide was giving a simulated drift and imaged every 30 seconds for 7 minutes. Left: convallaria was shifted by the simulated drift without correction. Right: Same drift was applied but the AutoCenter plugin corrected the drift during acquisition.

**Supplementary Video 3**

The cassette coated with green fluorescing polymer beads was stretched for 120 minutes. Images were taken every 30 seconds.

**Supplementary Video 4**

Two stage 13 *Xenopus laevis* animal cap organotypic explants were stretched and imaged on an inverted brightfield microscope with a 2.5x objective. Images were taken every 18 seconds for 87 minutes.

**Supplementary Video 5**

A stage 13 *Xenopus laevis* animal cap organotypic explant expressed with membrane-mNeonGreen was stretched and imaged on an inverted spinning disk confocal microscope with a 25x water-immersion objective. Images were taken for 8 stretch steps every two minutes over 16 minutes in total.

**Supplementary Video 6**

Mouse neonatal cardiomyocytes were imaged after stretching on an inverted spinning disk confocal microscope with a 63x oil-immersion objective. Cardiomyocytes remained intact and continued to beat after stretching. The arrow indicated the membrane rupture site. Images were taken every 15 seconds for 10 minutes. Magenta: membrane; cyan: nuclei.
